# RNAi-mediated control of CRISPR functions

**DOI:** 10.7150/thno.44880

**Published:** 2020-05-17

**Authors:** Xinbo Huang, Zhicong Chen, Yuchen Liu

**Affiliations:** 1 National and Local Joint Engineering Laboratory of Medical Synthetic Biology, Shenzhen, Second People's Hospital, The First Affiliated Hospital of Shenzhen University Health Science Center, Shenzhen518035, China; 2 Department of Urology, Shenzhen Second People's Hospital, The First Affiliated Hospital of Shenzhen University Health Science Center, Shenzhen518035, China; 3 Guangdong Key Laboratory of Systems Biology and Synthetic Biology for Urogenital Tumors, Shenzhen Second People's Hospital, The First Affiliated Hospital of Shenzhen University Health, Science Center, Shenzhen 518035, China

**Keywords:** CRISPR switch, artificial miRNA, miRNA sponge, enoxacin

## Abstract

CRISPR-Cas9 has become a versatile tool for genome editing and regulation, and strategies to effectively control its activity have attracted much attention. RNAi, also a gene-regulating tool, is used as another mechanism by which eukaryotes resist the invasion of foreign genetic material.

**Methods**: In this study, we analyzed the quantitative inhibition of the CRISPR system by using artificial miRNAs (amiRNAs) combined with the RNAi enhancer enoxacin to improve the targeting specificity of the CRISPR system. Furthermore, we examined the feasibility of improving the efficiency of gene editing and regulation by blocking the effects of natural intracellular miRNAs on sgRNAs.

**Results**: amiRNAs targeting the sgRNA were used to control its expression, and the small molecule drug denoxacin was utilized to enhance this effect, especially in the presence of Cas9. amiRNA/enoxacin inhibited CRISPR-mediated gene editing and regulation both *in vitro* and* in vivo* and could tune sgRNA-targeting specificity. Furthermore, CRISPR efficiency was increased by blocking the effects of endogenous miRNAs.

**Conclusion**: Our study provides an efficient molecular switch for conditional regulation of CRISPR activities in mammalian cells and also presents potentially useful approaches for solving current key issues of off-target effects and low targeting efficiency.

## Introduction

CRISPR-Cas9 has become a versatile tool for genome editing, gene regulation, and live imaging in a wide range of organisms 1, 2. The engineered CRISPR-Cas9 system contains two components: the Cas9 endonuclease and a single guide RNA (sgRNA), which recruits the Cas9 protein to a target DNA sequence 3. However, in some cases, the technology has not been proven to be accurate enough, with various unexpected off-target effects 4, 5. To render CRISPR-based editing more precise and safer, strategies to control CRISPR-Cas9 activity are highly desired. The tools to effectively and reversibly control the activity of CRISPR systems can alleviate safety concerns related to their accidental misuse. The newly developed CRISPR switches based on small molecules 6, 7 or light 8, 9 have attracted much attention. By adding exogenous inducers, the expression of the CRISPR system or the recombination of the Cas9 functional domains can be controlled to achieve the spatiotemporal specificity of gene editing. However, approaches necessary for inducing gene expression may have problems such as low induction efficiency and delayed gene expression. The strategy of splitting Cas9 protein may reduce the background efficiency of the system. The weak penetration of light into tissues is also a problem worth considering. Synthetic circuits controlling CRISPR expression are another area of active interest 10, 11. These methods may be limited by the complexity of the genetic circuits and the rare types of control nodes that can be rewired to control CRISPR systems. Recently, phage-encoded ''anti-CRISPR'' proteins 12-14 have been shown to block Cas9-mediated gene editing and regulation in bacterial and human cells. However, this approach requires the introduction of a foreign protein, and would broadly inhibit all sgRNA/Cas9 complexes within cells. To this end, methods for controlling CRISPR systems have been developed, but there is still a need for new tools for more specific, precise, and efficient control of the CRISPR system.

RNA interference (RNAi) is a well-conserved mechanism that uses small noncoding RNAs, such as small interfering RNAs (siRNAs) and microRNAs (miRNAs), to silence gene expression post-transcriptionally 15. Mammalian miRNAs are endogenous 20-25-nucleotide (nt) RNA guides that mediate mRNA degradation by pairing with the mRNAs of protein-coding genes 16. The most conserved motif pairs with nucleotides between 2 and 7 at the 5' end of the miRNA, which is called the 'seed' region, and the mRNA binding site. The functional unit of miRNA-mediated RNAi forms a complex with Argonaute proteins known as the miRNA-induced silencing complex (miRISC) 17. The mammalian miRNAs can target not only protein-coding mRNAs in the cytoplasm, but also regulate noncoding RNAs in the nucleus 18, 19. A small-molecule enoxacin (Penetrex) was found to enhance RNAi by promoting the processing and loading of miRNAs onto the miRISC 20, 21. Besides natural miRNAs, artificial miRNAs (amiRNAs) were also developed by several laboratories using natural miRNA scaffolds to target unusual RNAs 22. amiRNA-based approaches do not disrupt native cellular processes and may provide safer RNAi expression vectors compared with short hairpin RNAs (shRNAs).

Both RNAi and CRISPR systems cleave their targets using nucleases mediated by the guide sequences. Although RNAi and CRISPR-Cas9 have many similarities in terms of their mechanisms of action, few studies have suggested a direct relationship between the two in mammalian cells. Considerable evidence has suggested a role of mammalian miRNAs in restricting exogenous viral nucleic acids 23, 24. Therefore, it would be interesting to study whether mammalian miRNAs can block the foreign CRISPR system. Two studies have suggested that control of the CRISPR system using mammalian miRNAs can be achieved by inserting the miRNA binding sequence into the 5'UTR of Cas9 mRNA 25 or the 5' and 3' ends of the sgRNA 26. However, previous studies have ignored the potential impact of RNAi on the wild-type sgRNAs in mammalian cells. Although it is known that Cas9/sgRNA are localized in the nucleus and that miRISCs are mainly localized in the cytoplasm, miRNAs may regulate sgRNAs directly by returning to the nucleus to act on noncoding RNAs 18, 19.

We hypothesized that miRNAs could exert an inhibitory effect on the CRISPR system's function by binding to sgRNAs. We studied the quantitative inhibition of the CRISPR system by miRNAs combined with the RNAi enhancer enoxacin and attempted to improve the targeting specificity of the CRISPR system. Furthermore, we studied the feasibility of improving the efficiency of gene editing and regulation by blocking the effects of natural intracellular miRNAs on sgRNAs. The results revealed the competitive relationship between the RNAi pathway and the CRISPR system at the sgRNA level. Thus, our study represents a novel approach for resolving key issues of CRISPR research, including spatio-temporal specific regulation of gene editing or regulation, improving sgRNA targeting specificity, and enhancing the function of the CRISPR system.

## Results

### Effects of amiRNAs on the expression of sgRNAs

**Figure 1:**
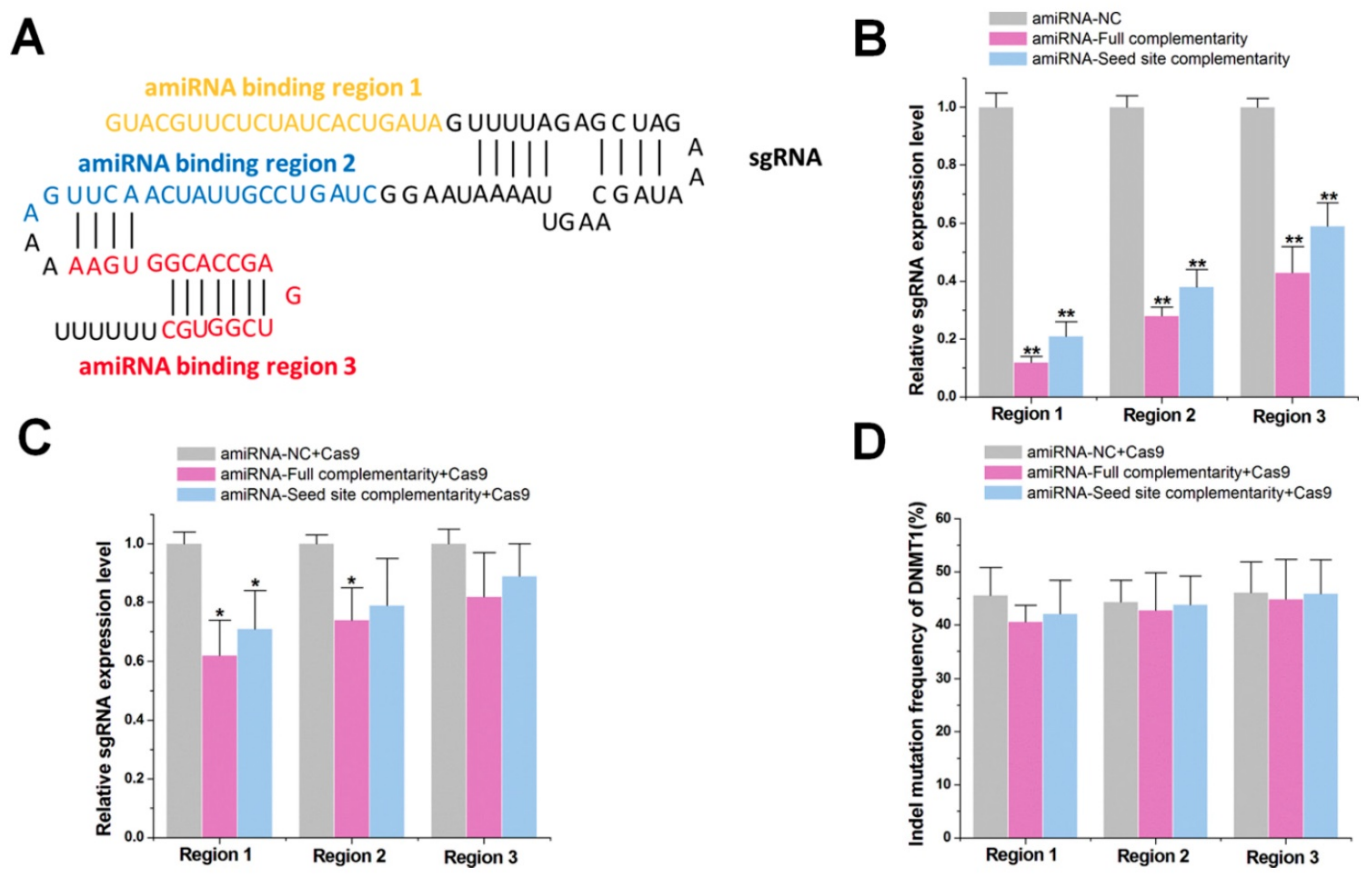
** Effects of amiRNAs on the expression of sgRNAs**. (**A**) Binding sites of amiRNAs in different regions of sgRNA. Region 1 represents the spacer sequence, and regions 2 and 3 are located in the sgRNA backbone. (**B**) Effects of amiRNAs on the expression of naked sgRNA by qRT-PCR. *GAPDH* was used as a control. Data are the mean ± SD from five experiments. ^**^P < 0.01, compared with the amiRNA negative control using the paired, one-sided *t*-test. (**C**) Effects of amiRNAs on the expression of sgRNA protected by Cas9 protein. *GAPDH* was used as a control. Data are the mean ± SD from five experiments. ^*^P < 0.05, compared with the amiRNA negative control using the paired, one-sided *t*-test. (**d**) Effects of amiRNAs on Cas9-mediated *DNMT1* cleavage efficiency. Data are the mean ± SD from five experiments.

To investigate the impact of the RNAi pathway on the CRISPR system, we first tested whether amiRNAs reduced sgRNA expression by targeting different sgRNA sites. We developed an amiRNA expression vector, which carried the designed mature miRNA sequences that were embedded within a widely used miRNA scaffold (miR-30) and were driven by the CMV-Pol II promoter. This design approach could efficiently generate highly active amiRNAs (Figure S1 A-C). We constructed amiRNAs complementary to three different regions of the negative control sgRNA sequence (non-targeting sgRNA sequence) that either bound to the spacer or to the sgRNA backbone (Figure 1A). The designed mature amiRNA sequences contained either a complementary seed sequence (6 nts) or a full complementary sequence (20 nts). A 6 nt sequence to mediate RNAi was used because miRNA target recognition is primarily determined by the pairing of its seed sequence (nucleotides 2-7) to complementary match sites in each RNA target 27, 28. Human embryonic kidney 293T (HEK-293T) cells were co-transfected with plasmids encoding amiRNAs and the sgRNA. After 48 h, quantitative real-time PCR (qRT-PCR) results indicated that amiRNAs targeting the sgRNA demonstrated effective repression, whereas those targeting the sgRNA backbone showed weaker effects (Figure 1B), possibly due to the low accessibility of hairpin structures to amiRNAs. Furthermore, amiRNAs with seed site complementarity also led to slightly less sgRNA degradation.

We next tested whether amiRNAs could also reduce sgRNA expression in the presence of Cas9 protein. Plasmids encoding amiRNAs and the sgRNA/Cas9 complex were co-transfected into HEK-293T cells. At 48 h, qRT-PCR results revealed that the inhibitory effect of each miRNA was decreased after the addition of Cas9 protein (Figure 1C), possibly due to the binding of Cas9 to sgRNA, which prevented the degradation mediated by amiRNA. To further test whether the amiRNAs inhibited the gene cleavage efficiency of the sgRNA/Cas9 complex, we redesigned the amiRNAs to target the spacer sequence of sgRNA-*DNMT1*. We found that amiRNAs did not reduce indel mutation rates for *DNMT1* at 48 h after transfection with the sgRNA/Cas9 complex using PCR and TIDE analyses (Figure 1D). Increasing the concentration of the transfected amiRNA caused cell death (data not shown). Similar results were observed with sgRNA-*DNMT1* when we examined its expression level regulated by amiRNAs (Figure S2A-B). Taken together, these results suggested that amiRNAs did not significantly affect the function of the sgRNA/Cas9 complex, although they effectively inhibited the expression of naked sgRNA.

### Initiation and enhancement of amiRNA-mediated CRISPR inhibition by enoxacin

**Figure 2:**
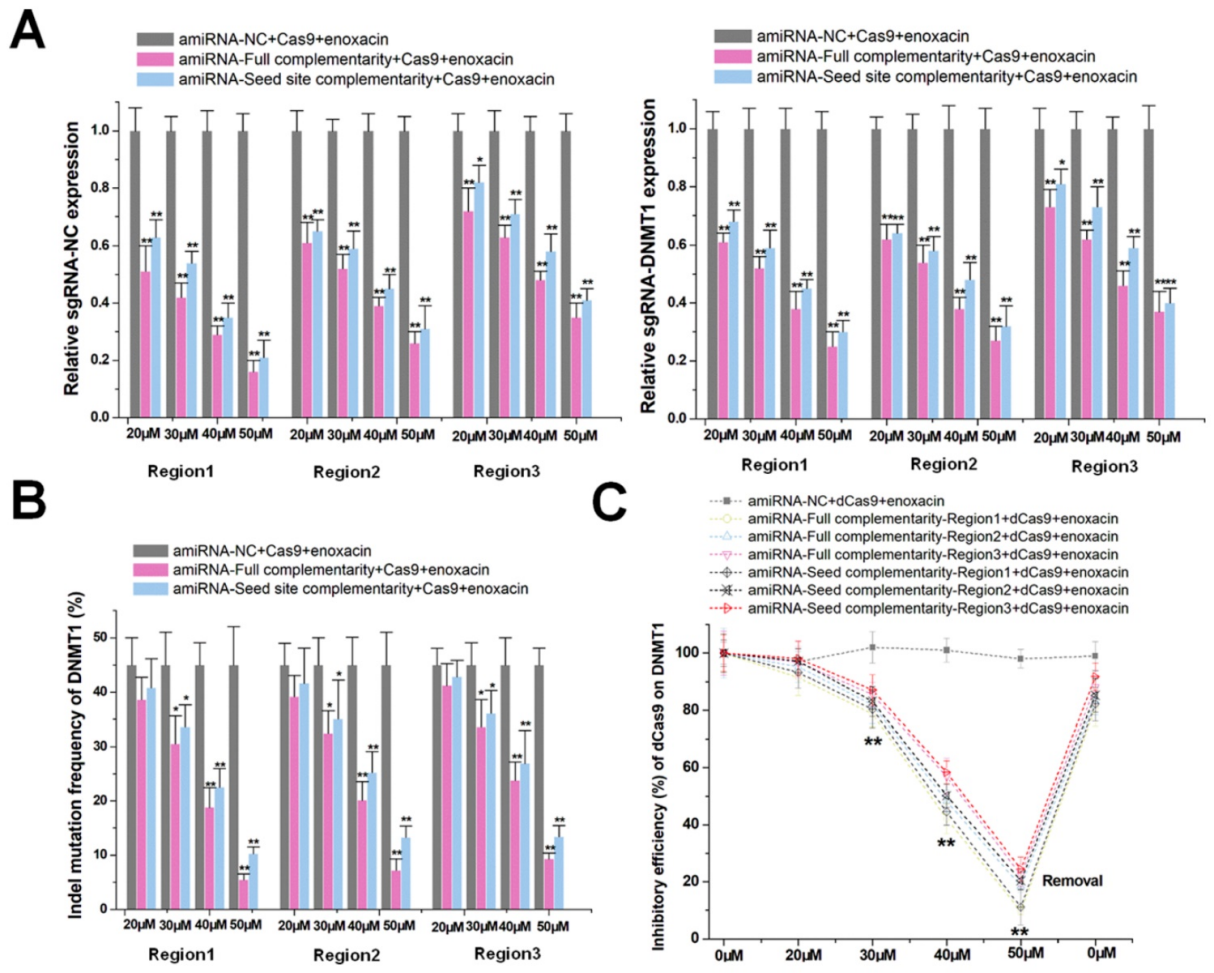
** Enoxacin promotes amiRNA-mediated CRISPR inhibition**. (**A**) Effects of amiRNAs on the expression of sgRNA at different enoxacin concentrations. amiRNA-NC, negative control amiRNA designed with no known RNA target in cells. sgRNA-NC, negative control sgRNA designed with no target gene in the human genome. Data are the mean ± SD from five experiments. ^**^P < 0.01, compared with the negative control using the paired, one-sided *t*-test. ^*^P < 0.05, compared with the negative control using the paired, one-sided *t*-test. (**B**) Effects of amiRNAs on Cas9-mediated *DNMT1* cleavage efficiency at different concentrations of enoxacin. Data are the mean ± SD from five experiments. ^**^P < 0.01, compared with the amiRNA negative control using the paired, one-sided *t*-test. ^*^P < 0.05, compared with the amiRNA negative control using the paired, one-sided *t*-test. (**C**) Effects of amiRNAs on dCas9-mediated *DNMT1* transcriptional suppression at different enoxacin concentrations. Data are the mean ± SD from five experiments. ^**^P < 0.01, compared with the amiRNA negative control using the paired, one-sided *t*-test.

The use of amiRNA alone did not effectively inhibit the function of the CRISPR-Cas9 system. We, therefore, used an RNAi enhancer enoxacin 20, 21 to assist amiRNA in inducing RNAi. After co-transfection of sgRNA/Cas9 with amiRNAs in HEK-293T cells, enoxacin initiated and increased amiRNA-mediated sgRNA-negative control and -*DNMT1* repression in a dose-dependent manner as indicated by qRT-PCR (Figure 2A). In contrast, the negative control of amiRNA, which did not recognize any RNA target, had no effect on the sgRNA expression. We used 50 μM of enoxacin, as a higher concentration could cause cell death (Figure S3A) and a concentration below 50 μM had no effect on Cas9 expression (Figure S3B). To further confirm that this effect was primarily driven by stoichiometry, we changed the relative transfection ratio of sgRNA/Cas9 and amiRNA, and found that the silencing effect of enoxacin increased with the decrease of sgRNA/Cas9 (Figure S4A) and with the increase of amiRNA (Figure S4B). The use of the amiRNAs with seed site complementarity also led to less decline in sgRNA degradation compared to amiRNAs with full sgRNA complementarity. Furthermore, amiRNAs targeting the sgRNA backbone showed weaker effects on sgRNA repression. For amiRNA binding to the spacer sequence of sgRNA-*DNMT1*, the possible effects on *DNMT1* mRNA expression were also examined. The mRNA level was almost unaffected in the presence of enoxacin (Figure S5), indicating that most intracellular amiRNAs bound to sgRNAs.

To determine the potential mechanism of amiRNAs/enoxacin-mediated sgRNA repression, amiRNA targeting the spacer sequence of sgRNA-negative control was used as a model. At 48 h after transfection, proteins extracted from HEK-293T were immunoprecipitated with Cas9 antibody, and qRT-PCR was used to detect the binding of sgRNA to the Cas9 protein. As the concentration of enoxacin increased, the binding rate of sgRNA to Cas9 decreased rapidly (Figure S6), suggesting that amiRNA may compete with Cas9 for binding to sgRNA. The effect of enoxacin on the processing and loading of amiRNAs was also determined by qRT-PCR. The addition of enoxacin moderately increased the number of amiRNAs, but no significant gradient effect was observed (Figure S7A). Next, Ago2-containing miRISCs were isolated through immunoprecipitation from HEK-293T. The number of amiRNAs associated with Ago2-containing miRISCs was proportionately increased upon treatment with enoxacin, suggesting that enoxacin enhanced amiRNA-mediated sgRNA inhibition mainly by promoting the loading of amiRNAs onto miRISCs (Figure S7B). Furthermore, the relative expression of sgRNA from Ago2-containing miRISCs was determined. The sgRNA could be detected in the amiRNA transfection group, and its relative expression level increased with increasing enoxacin concentration, while it could not be detected in the amiRNA-negative control transfection group (Figure S8). These results suggested that amiRNA in miRISCs could directly bind to sgRNA.

To further test whether amiRNA combined with enoxacin inhibited the DNA cleavage function of CRISPR-Cas9, we determined the indel mutation rates for *DNMT1* using PCR and TIDE analyses 48 h after transfection with sgRNA/Cas9. As shown in Figure 2B, the combination effectively inhibited the DNA cleavage function of Cas9 and exhibited a significant dose-dependent effect. Based upon these observations, we determined whether amiRNA/enoxacin combination could also be used to inhibit the transcriptional activity of a dead Cas9 (dCas9) system. We transfected HEK-293T cells with both dCas9 and *DNMT1* sgRNA and measured the CRISPRi-mediated regulation of *DNMT1* in response to different doses of enoxacin at 48 h post-transfection. The dose-effect curve suggested that the inhibition of amiRNA on CRISPRi was enhanced with increased enoxacin concentration (Figure 2C). Interestingly, the effect of CRISPRi was restored within 8 h when we removed enoxacin from the HEK-293T cell culture medium, indicating that amiRNA-enoxacin-mediated CRISPR inhibition was inducible and reversible. We also constructed a CRISPR-dCas9-VP64 transcriptional activation system, and obtained similar results by detecting the *DNMT1* transcript level (Figure S9). Together, the results suggested that the addition of RNAi enhancer enoxacin could achieve amiRNA-mediated CRISPR inhibition.

### amiRNA-mediated switching of CRISPR inhibitory patterns

**Figure 3:**
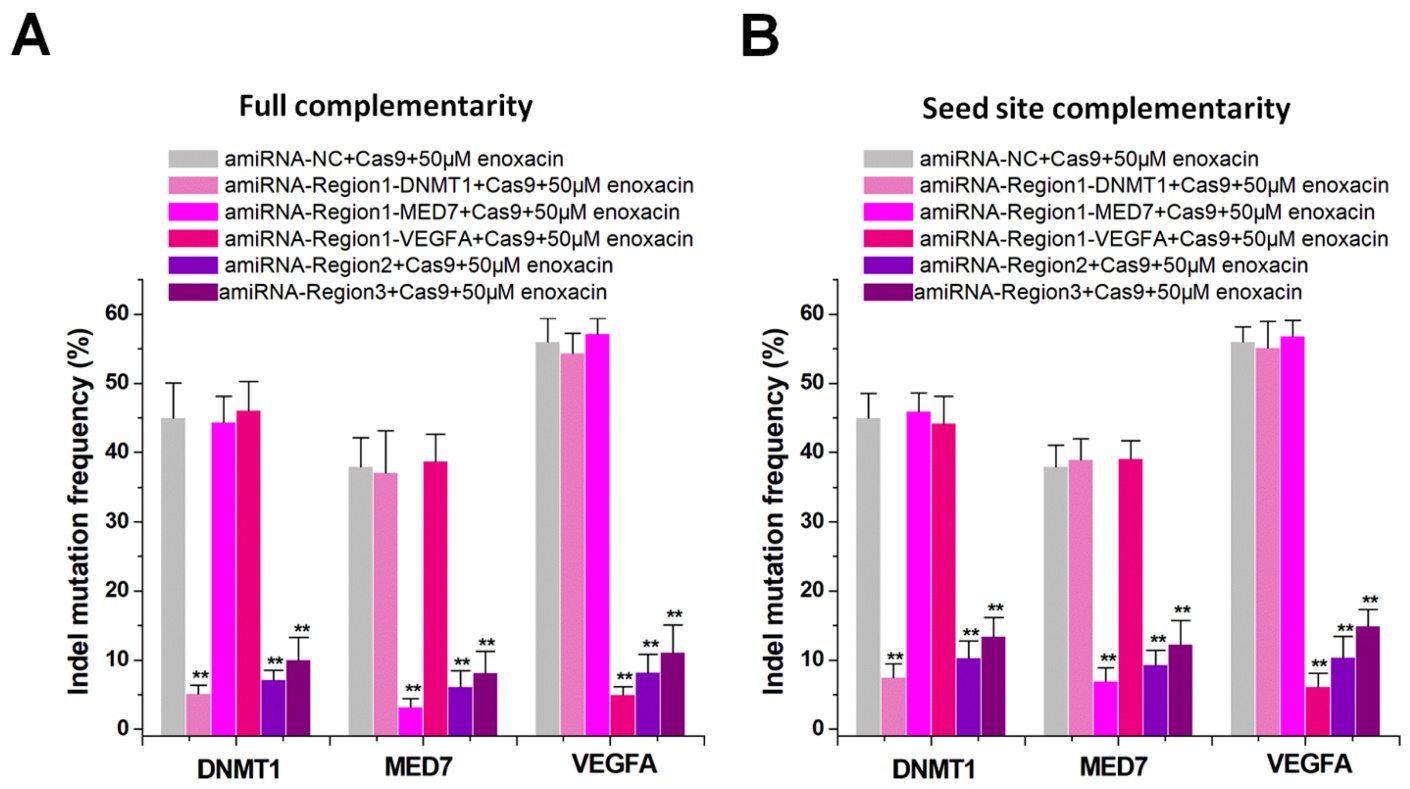
** Differential amiRNA-mediated CRISPR inhibition patterns.** (**A**) Effects of amiRNAs with full sgRNA complementarity on Cas9-mediated gene cleavage efficiency. Data are expressed as the mean ± SD. ^**^P < 0.01, compared with the negative control, determined with a paired, one-sided *t*-test. (**B**) Effects of amiRNAs with seed site complementarity on Cas9-mediated gene cleavage efficiency. Data are the mean ± SD from five experiments. ^**^P < 0.01, compared with the negative control, determined with a paired, one-sided *t*-test.

Current CRISPR inhibitors usually inhibit the overall activity of the CRISPR system 12-14, but cannot inhibit specific sgRNAs. We hypothesized that amiRNAs could either inhibit a specific sgRNA or all sgRNAs by targeting different sgRNA regions. We constructed multiple sgRNAs to target different genes such as *DNMT1*, *MED7*, and *VEGFA*, and designed different amiRNAs to target the sgRNA spacer or backbone. We determined the indel mutation rates using PCR and TIDE analyses after 48 h of transfection with sgRNA/Cas9. The amiRNA targeting sgRNA spacer in combination with enoxacin (50 μM) specifically inhibited one sgRNA, while the amiRNA targeting the sgRNA backbone inhibited all sgRNAs. The amiRNAs with full sgRNA complementarity (Figure 3A) showed a stronger inhibition of CRISPR function compared to those of amiRNAs with seed site complementarity (Figure 3B). In addition to the Cas9-mediated DNA cleavage system, we also used this strategy to control dCas9 transcriptional repression (Figure S10A-B) and dCas9-VP64 transcriptional activation (Figure S11A-B) systems and obtained similar results. These results suggested that amiRNA/enoxacin combination-mediated CRISPR inhibition could be switched from overall to specific inhibitory patterns.

### Inhibition of CRISPR function* in vivo* by amiRNA/enoxacin

**Figure 4:**
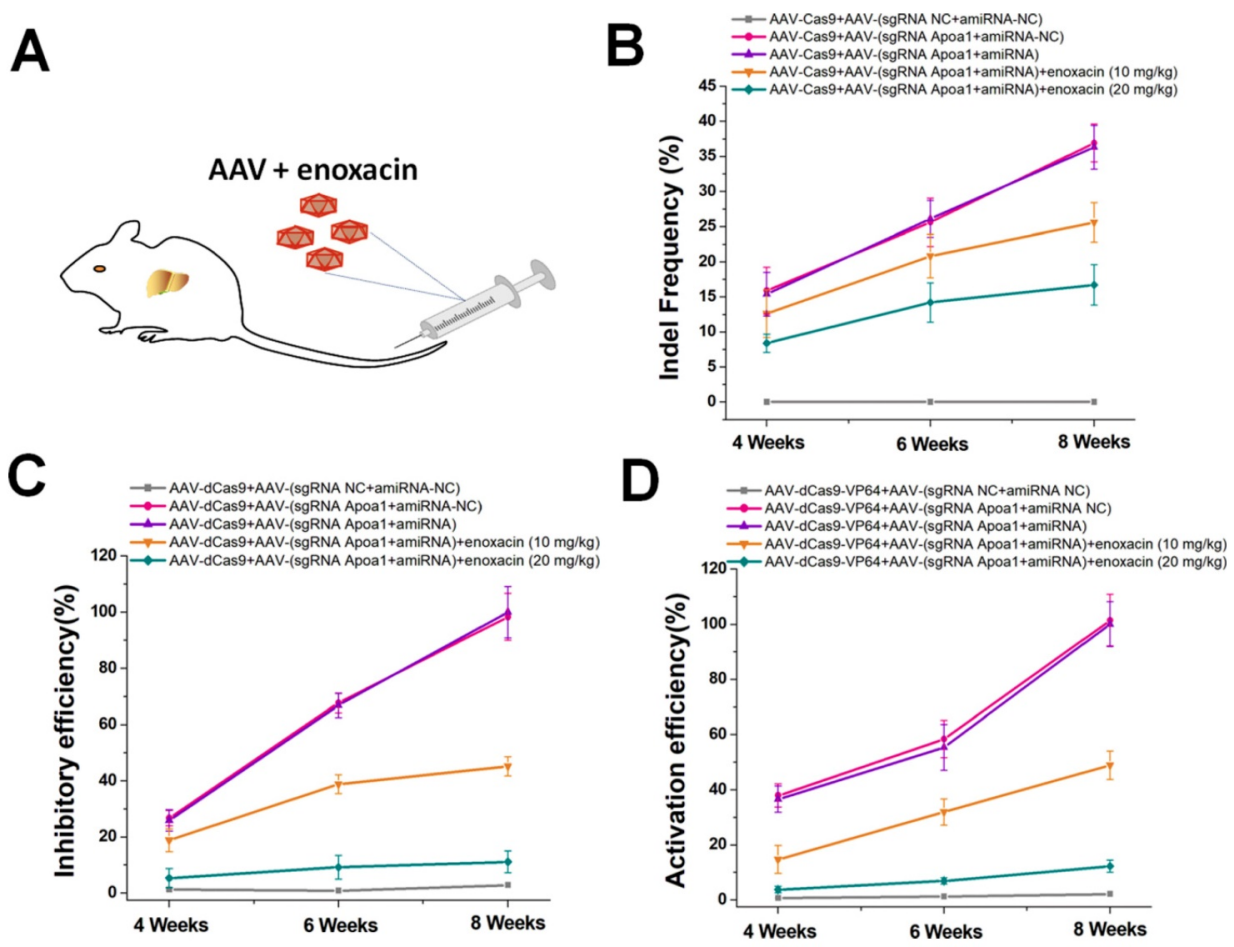
***In vivo* gene editing and regulation controlled by amiRNAs.** (**A**) AAV expressing the amiRNA or CRISPR system was injected via the tail vein of the mouse. (**B**) Effects of amiRNAs with full sgRNA complementarity on Cas9-mediated gene cleavage efficiency. Data are the mean ± SD from five experiments. (**C**) Effects of amiRNAs with full sgRNA complementarity on Cas9-mediated gene inhibitory efficiency. The *Apoa1* mRNA level was determined by qRT-PCR. Data are the mean ± SD from five experiments. (**D**) Effects of amiRNAs with full sgRNA complementarity on Cas9-mediated gene activation efficiency. The *Apoa1* mRNA level was determined by qRT-PCR. Data are the mean ± SD from five experiments.

The ideal inhibitory effect of the amiRNA/enoxacin combination on CRISPR *in vitro* prompted us to further investigate the ability of this strategy to regulate CRISPR functions *in vivo*. As an endogenous gene target for *in vivo* studies, we selected *Apoa1*, a hepatocyte-specific gene studied previously 29. AAV/CRISPR-mediated *Apoa1* editing and regulation controlled the liver lipid metabolism in mice. For targeted gene editing *in vivo*, we used AAV8 with high liver tropism to generate two different AAVs, one encoding Cas9 and the other encoding sgRNA-*Apoa1* and amiRNA fully complementary to the sgRNA-*Apoa1* spacer. We administered each AAV at a dose of 3 × 10^11^ viral genomes per vector per mouse (vg/v/m) by tail vein injection to C57Bl/6 mice with different doses of enoxacin (Figure 4A). To determine the inhibitory effects of amiRNA/enoxacin combination on CRISPR function, we determined indel mutation rates of *Apoa1* in the liver at 4, 6, and 8 weeks post-treatment. Compared with the amiRNA and sgRNA controls, we observed a greater inhibitory dose-dependent effect of amiRNA/enoxacin combination on CRISPR function in mouse livers (Figure 4B). Furthermore, we observed similar trends in transcriptional changes of *Apoa1* using AAV-dCas9/sgRNA (Figure 4C) and AAV-dCas9-VP64/sgRNA (Figure 4D). Together, our results indicated that the amiRNA/enoxacin approach inhibited CRISPR function *in vivo*.

### Reducing off-target events by amiRNA-mediated RNAi

**Figure 5:**
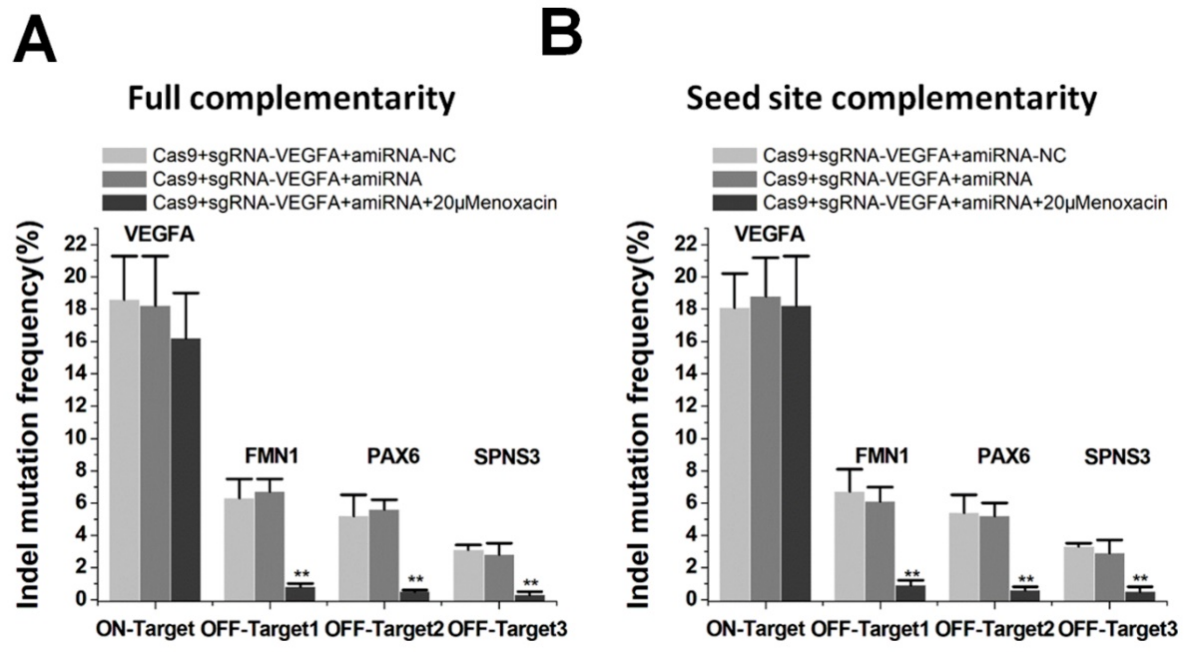
** Reduction of off-target events mediated by amiRNA.** (**A**) Effects of amiRNAs with full sgRNA complementarity on sgRNAs. Data are the mean ± SD from five experiments. ^**^P < 0.01, compared with the negative control, determined with a paired, one-sided *t*-test. (**B**) Effects of amiRNAs with seed site complementarity on sgRNA. Data are the mean ± SD from five experiments. ^**^P < 0.01, compared with the negative control, determined with a paired, one-sided *t*-test.

Studies have suggested that CRISPR/Cas9 systems caused serious off-target problems 4,5. The binding efficiency of sgRNA was different between on-target and off-target sites, and the off-target site may be insufficiently bound 30. We determined whether this property could be used to specifically inhibit Cas9-mediated cleavage at the off-target sites with amiRNAs. We examined on- and off-target effects using the sgRNA targeting *VEGFA*, as the three off-target sites for *VEGFA* have been previously described 31. We edited *VEGFA* loci using sgRNA/Cas9 with and without co-treatment of the amiRNA/enoxacin. TIDE analyses showed that the addition of amiRNA targeting sgRNA spacer with a low concentration of enoxacin (20 μM) significantly abolished off-target editing. The amiRNAs with full complementarity also had a weak inhibitory effect on the cleavage of sgRNA at the on-target site, but the difference was not statistically significant compared to controls (Figure 5A). Interestingly, amiRNAs with seed site complementarity had no effect on the editing efficiency of the on-target site with 20 μM enoxacin (Figure 5B). To further confirm the significance of this strategy for the inhibition of off-target effects, we used another *VEGFA* sgRNA that had multiple off-target sites, as previously reported 31 and obtained similar results (Figure S12A-B). To further demonstrate the mechanism by which amiRNAs reduced the off-target efficiency of CRISPR, we tested the editing efficiency of sgRNA/Cas9 on VEGFA in the presence of 50 μM enoxacin. The efficiencies of sgRNAs at both on-target and off-target sites were significantly inhibited by amiRNAs in the presence of 50 μM enoxacin (Figure S13A-B), indicating that the effect of amiRNAs on reducing the off-target efficiency of CRISPR could be explained by the dose effect of enoxacin. These results suggested that the amiRNA approach had the potential to reduce off-target events and could have therapeutic applications.

### Increasing CRISPR functions by miRNA sponges

**Figure 6:**
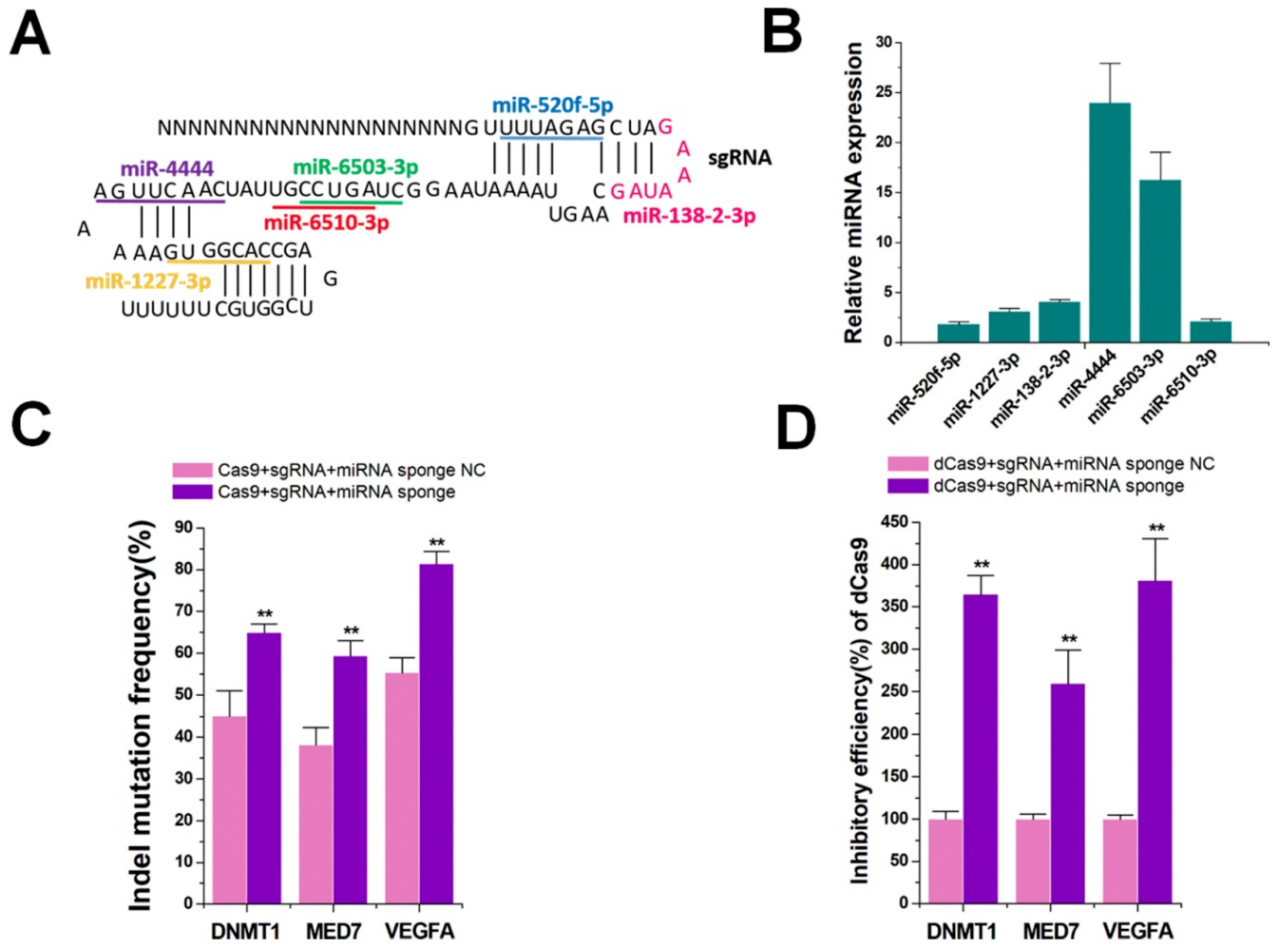
** Enhanced on-target efficiency by miRNA sponges.** (**A**) Binding sequence**s** of native miRNA**s** at different positions of the sgRNA. (**B**) Expression of different miRNAs in HEK-293T cells by qRT-PCR. U6 was used as an internal control. (**C**) Effects of miRNA sponge on Cas9-mediated gene cleavage efficiency. Data are the mean ± SD from five experiments. ^**^P < 0.01, compared with the negative control, determined with a paired, one-sided *t*-test. (**D**) Effects of miRNA sponge on dCas9-mediated transcriptional inhibition. Data are the mean ± SD from five experiments. ^**^P < 0.01, compared with the negative control, determined with a paired, one-sided *t*-test.

Besides targeting specificity, another important issue is of enhancing the efficiency of the CRISPR system 32. Although amiRNAs did not effectively inhibit the function of the sgRNA/Cas9 system without enoxacin, we determined if natural miRNAs could inhibit the function of CRISPR. The content and variety of natural miRNAs in cells are abundant, and their processing and action should be more effective than those of amiRNAs. We analyzed the sgRNA backbone of spCas9 using miRBase online predictive software (http://www.mirbase.org/), and found 6 target sites for different natural miRNAs (Figure 6A). We used qRT-PCR to detect the expression levels of these 6 miRNAs in HEK-293T cells. The results indicated that these miRNAs were all expressed to some extent in this cell line, among which miR-4444 and miR-6503-3p were most abundant (Figure 6B). To further determine whether these natural miRNAs affected CRISPR function, an miRNA sponge was designed to block the intracellular activity of these miRNAs. Six copies of the miRNA antisense sequence were designed to bind to miR-4444 or miR-6503-3p and the other four miRNAs were inhibited by 3 copies of the antisense sequence in the miRNA sponge. As a control, we constructed a sponge with 3 copies of repeated binding sites not complementary to any known miRNAs. As shown in Figure S14, the reduction of each miRNA through the addition of miRNA sponge was confirmed.

Next, we determined the indel mutation rates for *DNMT1, MED7*, and* VEGFA* using TIDE analyses 48 h after transfection with sgRNA/Cas9 and miRNA sponge. The results showed that the miRNA sponge increased the DNA cleavage function of CRISPR-Cas9 in HEK-293T cells (Figure 6C). The sgRNAs were also found to accumulate after treatment with miRNA sponges (Figure S15). To further confirm the universality of the miRNA sponge strategy, we utilized the dCas9 and dCas9-VP64 systems that inhibited (Figure 6D) and activated (Figure S16) three genes (*DNMT1*, *MED7* and *VEGFA*), yielding similar results. We also explored if CRISPR function could be inhibited by addition of enoxacin, which might enhance the RNAi efficacy of natural miRNAs. The results showed that even 50 μM enoxacin could not significantly increase the gene editing and regulation of the Cas9, dCas9, and dCas9-VP64 systems (Figure S17A-C). Thus, gene editing and regulation of the CRISPR system could be effectively improved after blocking natural miRNAs that bound to sgRNAs.

## Discussion

The RNAi and CRISPR pathways are highly specific and efficient RNA and DNA interference systems, respectively. The CRISPR system protects prokaryotes against phage infection, while RNAi is a potent antiviral system in eukaryotes 33, 34. Both systems are also widely used to screen and validate functional genes in many biological systems 35. CRISPR-Cas9 gene editing efficiency could be improved by reducing RNA silencing in plants 36. However, few studies focused on whether RNAi can directly regulate CRISPR function by regulating sgRNAs. As an immune defense system of eukaryotic cells, RNAi may provide a natural molecular switch to regulate CRISPR. One of the major advantages of using RNAi to regulate CRISPR is that it does not require the introduction of exogenous phage proteins into cells, avoiding the problems of expression efficiency, enzyme activity, and biosafety issues. RNAi, on the other hand, is an original and ancient mechanism of eukaryotic cell regulation, which has undergone a long evolutionary process and is very stable.

In this study, we provided direct evidence demonstrating the ability of RNAi to regulate the CRISPR system at the sgRNA level. The amiRNAs did not directly suppress sgRNA expression in the presence of Cas9 protein, probably because the sgRNA was protected by Cas9 and also due to the low efficiency of processing and loading amiRNAs. However, amiRNAs effectively repressed sgRNA/Cas9 binding and inhibited the efficiency of Cas9, both *in vitro* and *in vivo,* in the presence of the small molecular drug enoxacin. This allowed us to quantitatively regulate the activity of the CRISPR system by using varying concentrations of enoxacin. Moreover, this inhibition was reversible, providing a flexible strategy for controlling the activity of the CRISPR system.

**Figure 7:**
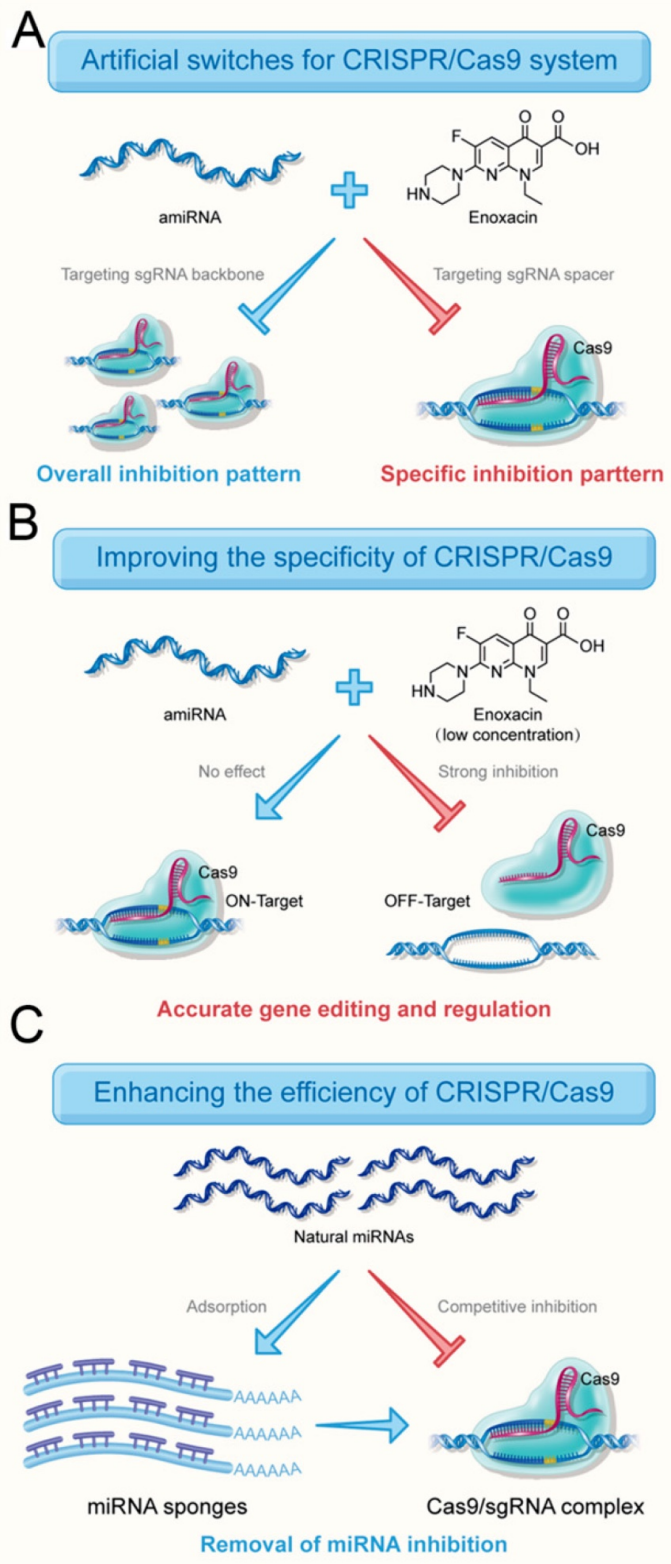
** Roles of RNAi-mediated CRISPR switches.** (**A**) amiRNAs inhibit the activity of the entire CRISPR system and also inhibit the activity of a specific sgRNA. (**B**) amiRNAs effectively reduce the off-target effects of Cas9 by targeting sgRNA spacers without affecting the editing efficiency of target genes in the presence of a low concentration of enoxacin. (**C**) amiRNAs increase the targeting efficiency by eliminating the effects of natural miRNAs with miRNA sponge

Compared to the existing CRISPR inhibitors, a major advantage of the amiRNA strategy is that CRISPR inhibition could be switched between overall and specific inhibitory patterns (Figure 7A) by choosing the targeting regions of amiRNAs. Especially for multi-gene editing and transcriptional regulation, only the sgRNA activity of a specific gene needed to be regulated and the amiRNA strategy perfectly met this requirement. This strategy may also apply to gene editing approaches that deliver sgRNA/Cas9 protein complexes directly into cells.

The potential off-target effects of the CRISPR system have been a major obstacle to CRISPR technology, because any off-target DNA cleavage can cause permanent and unexpected side effects and, therefore, attempts have been made to minimize this disadvantage. Improving fidelity is not only useful for gene functional studies, but also promotes its translational applications in the clinic 37. Also, the binding efficiency of sgRNA on an off-target site was reported to be much lower than that on the on-target site 30.

In the present study, we demonstrated that amiRNAs targeting sgRNA spacers could effectively reduce the off-target effects of Cas9 without affecting the editing efficiency of target genes in the presence of a low concentration of enoxacin (Figure 7B). This suggested that the perfect match between the sgRNA/Cas9 and the DNA target protected the sgRNA from interference by the RNAi system to some extent. The different inhibitory effects of amiRNAs targeting spacer region on sgRNA-*DNMT1*/Cas9 and sgRNA-NC/Cas9 (Figure 2A) also supported this contention. An amiRNA with seed site complementarity had no effect on cleavage of target DNA mediated by Cas9. It is of note that amiRNAs with seed site complementarity have less inhibitory effects on various CRISPR systems compared to amiRNAs with full sgRNA complementarity, suggesting that there is a competitive binding relationship between RNAi and CRISPR.

Finally, we tested natural miRNAs that inhibited the CRISPR system. Several natural miRNAs predicted by software to target sgRNA were found to be highly expressed in the HEK-293T cells. To remove the effects of natural miRNAs, we used the traditional strategy in which miRNAs were blocked by artificial sponges 38. The results confirmed that the miRNA sponge approach significantly improved the efficiency of gene editing and regulation of the CRISPR system (Figure 7C). A very interesting phenomenon was observed that natural endogenous miRNAs, but not amiRNAs, were able to inhibit sgRNA activity. The natural RNAi system is believed to have a direct inhibitory effect on the CRISPR system because the silencing of AGO might improve CRISPR efficiency 36. Therefore, we speculated that the reason for this difference was the low binding efficiency of amiRNAs and miRISCs, which was further enhanced by enoxacin.

Although CRISPR is generally effective, its editing efficiency between different organisms varies widely. Therefore, our study provides a new potentially useful way to improve the efficiency of CRISPR editing/regulation. Also, the effect of miRNA sponge strategy is likely much more specific than that of silencing AGO 36, because the knockdown of AGO may affect the entire RNAi interference system with a significant impact on the survival of cells. In the future, the combination of miRNA sponges and other CRISPR activating drugs 39, as well as the level of natural miRNAs that target sgRNA, should be considered before manipulating CRISPR. It is worth noting that a high concentration of enoxacin alone could not further enhance the inhibitory effect of natural miRNAs on CRISPR. We speculated that this might be because the cellular content of natural miRNAs was not as large as that of exogenously expressed amiRNAs, and/or the binding capacity of natural miRNAs to miRISC was inherently strong.

In summary, we reported the benefit of using enoxacin to regulate the CRISPR/Cas9 activity at the sgRNA level. The engineered regulatory system could achieve both reversible and selective suppression of specific sgRNAs by co-expressing corresponding amiRNAs in the presence of a small molecule drug enhancer. Moreover, we proposed that not only amiRNAs but also endogenous miRNAs could have an inhibitory role in sgRNA expression, which was not explored previously. Our study not only provides a novel and efficient molecular switch for conditional regulation of CRISPR activities in mammalian cells but also presents potentially useful approaches for solving current key issues of off-target effects and low targeting efficiency 40, 41.

## Materials and Methods

### Construction of the artificial miRNAs and miRNA sponges

The sequences of artificial miRNAs (or miRNA sponges) and their negative controls were designed and chemically synthesized. These synthetic elements were inserted into pcDNA3.1. All vectors were transformed into *Escherichia coli*, and the desired expression clones were identified by polymerase chain reaction (PCR) amplification followed by electrophoresis and were confirmed by sequencing. The sequences are presented in Tables S1 and S2.

### Design and construction of CRISPR plasmids

Human codon-optimized SpCas9 fused to an NLS (Addgene plasmid # 41815; Cambridge, MA, USA) was used to construct natural Cas9 and mutant dCas9 expression cassettes. A dCas9-VP64 fusion protein consisting of the synthetic VP64 activation domain linked to the C terminus of dCas9 was constructed. The sgRNAs were designed using the online design tool “CRISPR-ERA” (http://CRISPR-ERA.stanford.edu). The sequence information is shown in Table S3. All vectors were also transformed into *E. coli* cells, and the desired expression clones were identified using PCR amplification and electrophoresis, and subsequently confirmed with Sanger sequencing.

### Cell culture and cell transfection

HEK-293T cells were purchased from American Type Culture Collection (Manassas, VA, USA) and maintained in DMEM supplemented with 10% fetal bovine serum (Invitrogen, Carlsbad, CA, USA) in the presence of 5% CO_2_ at 37 °C in an incubator. For transient transfection experiments, cells were treated with the mixtures of plasmids using Lipofectamine 2000 Transfection Reagent (Invitrogen) according to the manufacturer's instructions. 2 × 10^ 5^ cells were co-transfected with 250 ng of sgRNA/Cas9 expression plasmid and 750 ng of amiRNA/miRNA sponge expression plasmid. Enoxacin was added to the cell culture medium 6 h after transfection.

### Dual-luciferase reporter assay

Luciferase activity was measured in a 1.5 mL Eppendorf tube with the Dual-Luciferases Reporter Assay kit (Promega, Madison, WI) according to the supplier's instructions 48 h post-transfection. Relative Renilla luciferase (Rluc) activity was determined as the value normalized to firefly luciferase (Fluc) activity. The assays were performed in duplicate, and the experiments were repeated three times.

### Green fluorescence observation

HEK-293T cells were transfected with the plasmids and then examined for GFP expression 48 h post-transfection using fluorescence microscopy (MicroPublisher 3.3 RTV; Olympus, Tokyo, Japan). Images were captured in the auto-exposure mode.

### Cell viability assay

Cell viability was measured using the CCK-8 assay. The HEK-293T cells were plated at a density of 1 × 10^3^ cells per well in a 96-well plate. After enoxacin treatment, 10 μl of the CCK-8 solution was added to cells in each well, followed by incubation for 2 h. Cell proliferation/viability was measured by determining the OD at 450 nm using a microplate reader. All treatments were measured in triplicate wells and repeated three times. Percent over untreated control was calculated as a measure of cell viability.

### Adeno-associated virus (AAV) packaging, purification, and infection

The pAAV packaging DNA construct, pHelper construct, and pAAV construct were co-transfected into HEK-293T cells using Lipofectamine 2000. The culture supernatants were collected at 48 h after transfection, concentrated, and used as virus stocks for the following AAV infection experiment. The AAV titer was calculated by qPCR using 2× EvaGreen Master Mix (Syngentech).

### RNA extraction and qRT-PCR

Tissue samples were stored in RNALater (Ambion, Austin, TX, USA), and total RNA was extracted from HEK-293T cells or liver tissues using TRIzol reagent (Invitrogen, Carlsbad, CA, USA) according to the manufacturer's protocols. The concentration and purity of total RNA were measured using UV spectrophotometric analysis at 260 nm. The cDNAs were synthesized using a Revertra Ace qPCR RT Kit (Toyobo, Osaka, Japan). Real-time PCR was carried out with real-time PCR Master Mix (Toyobo). *GAPDH* was selected as the endogenous control. The PCR mixtures were prepared according to the manufacturer's protocols and amplification was performed using PCR conditions of 40 cycles of 15 s at 95 °C, 20 s at 55 °C, and 30 s at 70 °C using an ABI PRISM 7300 Fluorescent Quantitative PCR System (Applied Biosystems, Foster City, CA, USA). The qPCR primer sequences of genes are presented in Table S4. Expression fold-changes were calculated using the 2^-△△ct^ method. The miRNAs were detected using the All-in-One^TM^ miRNA qRT-PCR Detection Kit (GeneCopoiea Inc, Rockville, MD, USA). U6 small nuclear RNA (snRNA) was selected as the endogenous control. The miRNA qPCR Primers were obtained from GeneCopoeia, Inc. The inhibitory efficiency (%) was determined by the formula: 100% × (relative mRNA expression level in the negative control group - relative mRNA expression level in the experimental group)/relative mRNA expression level in the negative control group. The activation efficiency (%) was determined by the formula: 100% × (relative mRNA expression level in the experimental group - relative mRNA expression level in the negative control group)/relative mRNA expression level in the negative control group.

### Determination of NHEJ-mediated indel mutations

The genomic DNA was extracted from transfected cells or mouse livers using the QuickExtract DNA Extraction system (Epicentre, Madison, WI, USA). PCR was then performed to amplify the target regions using the genomic DNA as the template. The PCR products were purified using the ISOLATE II PCR and Gel Kit (Bioline, Memphis, TN, USA), and Sanger sequenced. Total NHEJ frequencies were further calculated by decomposition of the sequencing chromatogram using the TIDE, an interactive software program (https://tide-calculator.nki.nl/), as described previously 42. Depicted values were generated from TIDER analyses with R^2^ values > 0.9 and P < 0.001.

### Immunoprecipitation of Cas9-sgRNA or Ago2-miRNA

48 h after transfection, cells were harvested and 2 × 10 ^5^ cells were extracted in 0.5 ml 50 mM Tris-HCl pH 8/150 mM NaCl/0.1% NP40 for immunoprecipitation. Clarified lysates were subjected to immunoprecipitation by incubation for 1.5 h at 4 °C with the Cas9 antibody (#ab191468, Abcam, MA) or Ago2 antibody (Sigma-Aldrich, SAB4200085). After the antibody was recovered by protein A/G beads, the Cas9/Ago2-bound RNA was extracted and subjected to qRT-PCR analysis.

### *In vivo* gene editing & regulation in mice

All experiments involving animals were approved by the Institutional Review Board. Animals were housed and handled in accordance with protocols. Four-week-old C57Bl/6 mice were obtained from the Animal Center of the Academy of Sciences. For each experiment, 75 age-matched mice by date of birth were assigned randomly to a treatment group (n=15 for each group), and injected with AAV solution (3 × 10^11^ vg/v/m /total dose) and enoxacin via the tail vein using a 31-gauge needle. The treatments by tail vein injections were repeated at a frequency of once every 2 weeks. Mice were euthanized at 4, 6, and 8 weeks (n=5 for each time point in each group) after the AAV injection, and liver specimens were harvested and processed for *Apoa1* gene editing efficiency and expression analysis.

### Statistical analyses

No statistical methods were used to predetermine the sample size. Statistical analyses were conducted using the *t*-test or analysis of variance, and a P <0.05 was considered statistically significant. All statistical tests were performed by SPSS statistical software for Windows, version 19.0 (SPSS, Chicago, IL, USA).

## Supplementary Material

Supplementary figures and tables.
