# Supplementary material for: RNAi-mediated control of CRISPR functions
Source: Theranostics. 2020 May 17;10(15):6661–73. doi: 10.7150/thno.44880 (PMC7295050; doi:10.7150/thno.44880)
Supplement: Supplementary file 1 — Supplementary figures and tables. [file thnov10p6661s1.pdf]

Supplementary figures and tables.

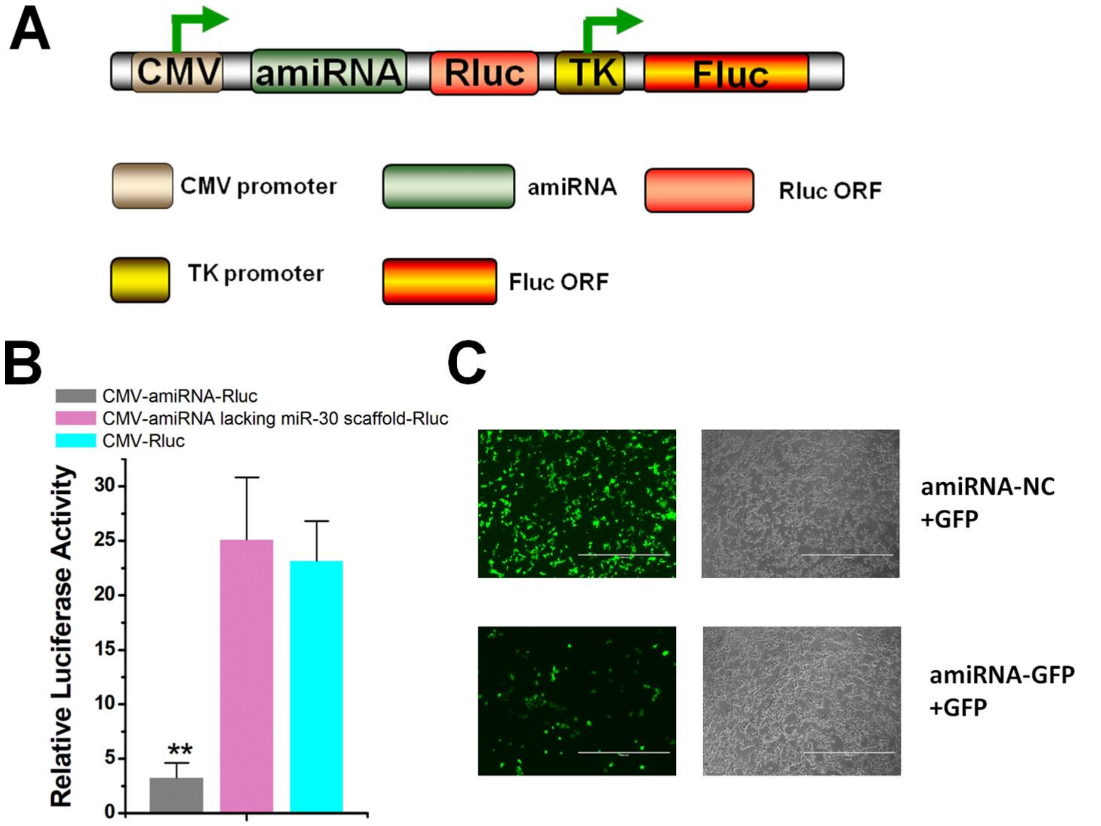

Figure S1. Detection of the activity of the generated amiRNAs. (**A**) The amiRNA targeting GFP was inserted into the 5' UTR of the renilla luciferase (Rluc) mRNA. Another TK promoter-driven firefly luciferase (FLuc) coexpressed on the same vector was used as an internal control. Rluc could not be efficiently translated because the processing of the miRNA led to a cleaved mRNA product that should be degraded quickly. (**B**) Compared with the constructs containing the control sequence, the ratio of Rluc / Fluc in the vector containing the amiRNA sequence was reduced in HEK-293T cells at 48h post-transfection. **P < 0.01, compared with the negative controls using the paired, one-sided t-test. (**C**) The green fluorescence in the amiRNA and GFP co-transfection group was also significantly lower than that in the amiRNA negative control and GFP co-transfection group at 48h post-transfection.

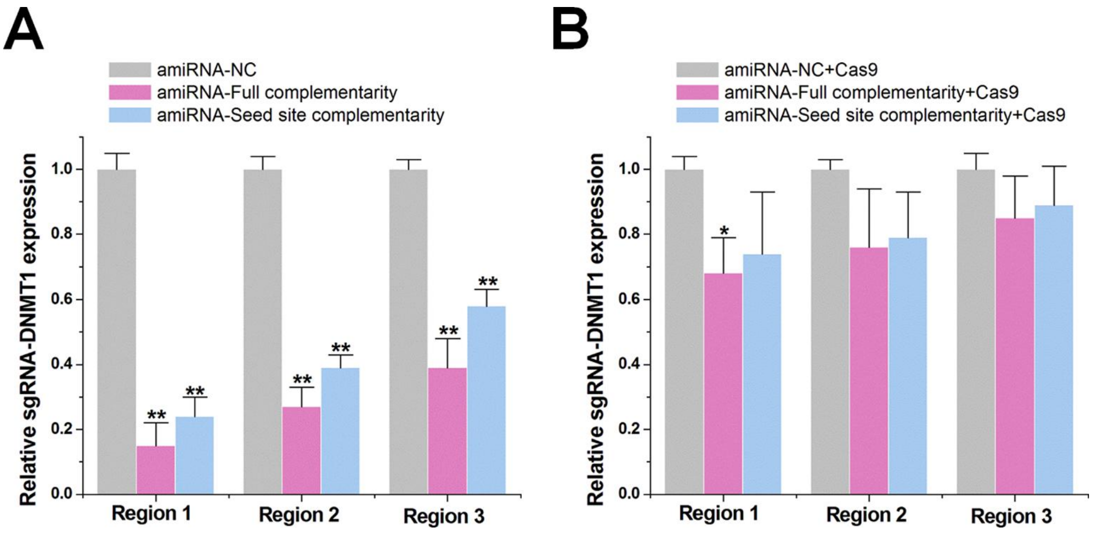

Figure S2. Effects of amiRNAs on the expression of sgRNA-*DNMT1*. (**A**) Effects of amiRNAs on the expression of naked sgRNA-*DNMT1* as revealed by the qRT-PCR assay. *GAPDH* was used as a control. Reported data are the mean ± SD from five experiments. **P < 0.01, compared with the amiRNA negative control using the paired, one-sided t-test. (**B**) Effects of amiRNAs on the expression of sgRNA-*DNMT1* protected by Cas9 protein. *GAPDH* was used as a control. Reported data are the mean ± SD from five experiments. *P < 0.05, compared with the amiRNA negative control using the paired, one-sided t-test.

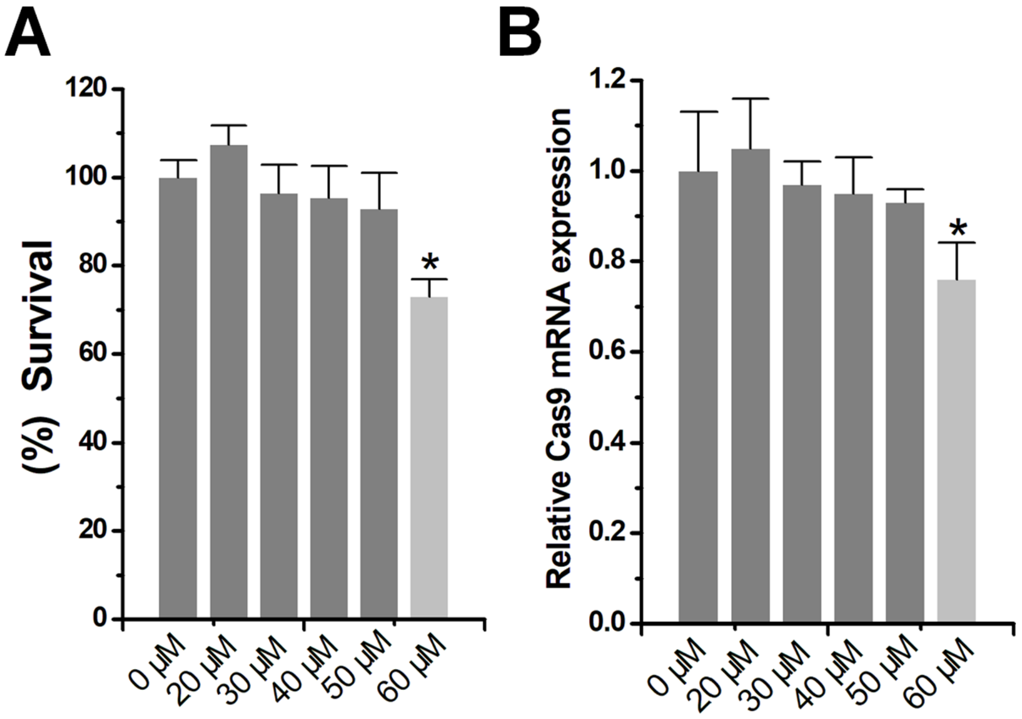

Figure S3. The effects of enoxacin on cell growth and Cas9 mRNA expression. (**A**) HEK-293T cells were treated with different concentrations of enoxacin for 48h, and cell viability was measured by CCK-8 assay. *$P < 0.05$, compared with the blank group using the paired, one-sided t-test. (**B**) HEK-293T cells were treated with different concentrations of enoxacin for 48h, and Cas9 mRNA expression was measured by qRT-PCR assay. *$P < 0.05$, compared with the blank group using the paired, one-sided t-test.

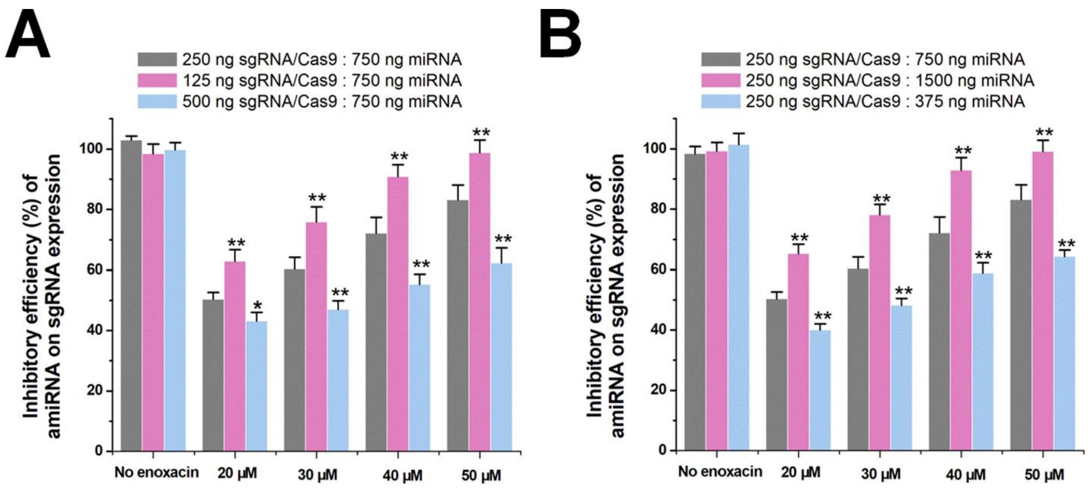

Figure S4. The inhibitory efficiency (%) of amiRNA on sgRNA expression under different ratios of sgRNA/Cas9 and amiRNA. sgRNA expression was measured by qRT-PCR assay. The inhibitory efficiency (%) was determined by the formula: 100% × (relative sgRNA expression level in the amiRNA negative control group - relative sgRNA expression level in the amiRNA group) / relative sgRNA expression level in the amiRNA negative control group. Data are the mean ± SD from five experiments. (**A**) The inhibitory efficiency (%) of amiRNA on sgRNA expression under different concentrations of sgRNA/Cas9. $^{**}P < 0.01$, compared with the control (250ng sgRNA/Cas9) using the paired, one-sided *t*-test. $^{*}P < 0.05$, compared with the control (250ng sgRNA/Cas9) using the paired, one-sided *t*-test. (**B**) The inhibitory efficiency (%) of amiRNA on sgRNA expression under different concentrations of amiRNA. $^{**}P < 0.01$, compared with the control (750ng amiRNA) using the paired, one-sided *t*-test.

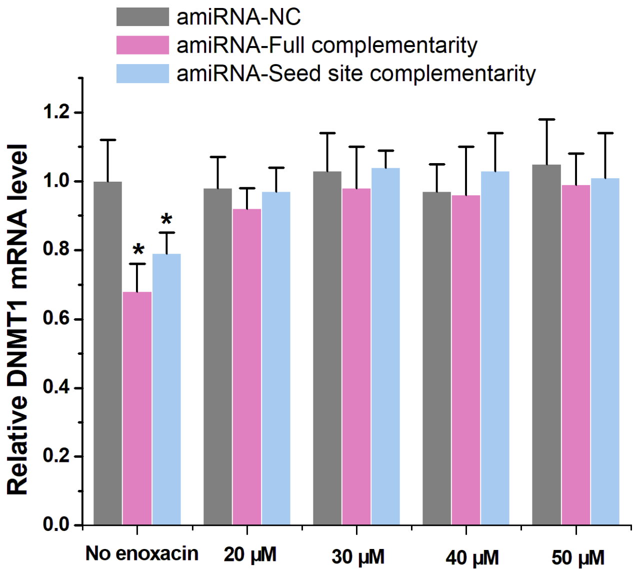

Figure S5. Effects of amiRNAs on the expression of *DNMT1* mRNA . Effects of amiRNAs on the expression of *DNMT1* mRNA in the presence of different concentrations of enoxacin was determined by the qRT-PCR assay. amiRNA, sgRNA-*DNMT1* and Cas9 protein were co-expressed in HEK-293T cells. *GAPDH* was used as a control. Reported data are the mean ± SD from five experiments. *P < 0.05, compared with the amiRNA negative control using the paired, one-sided t-test.

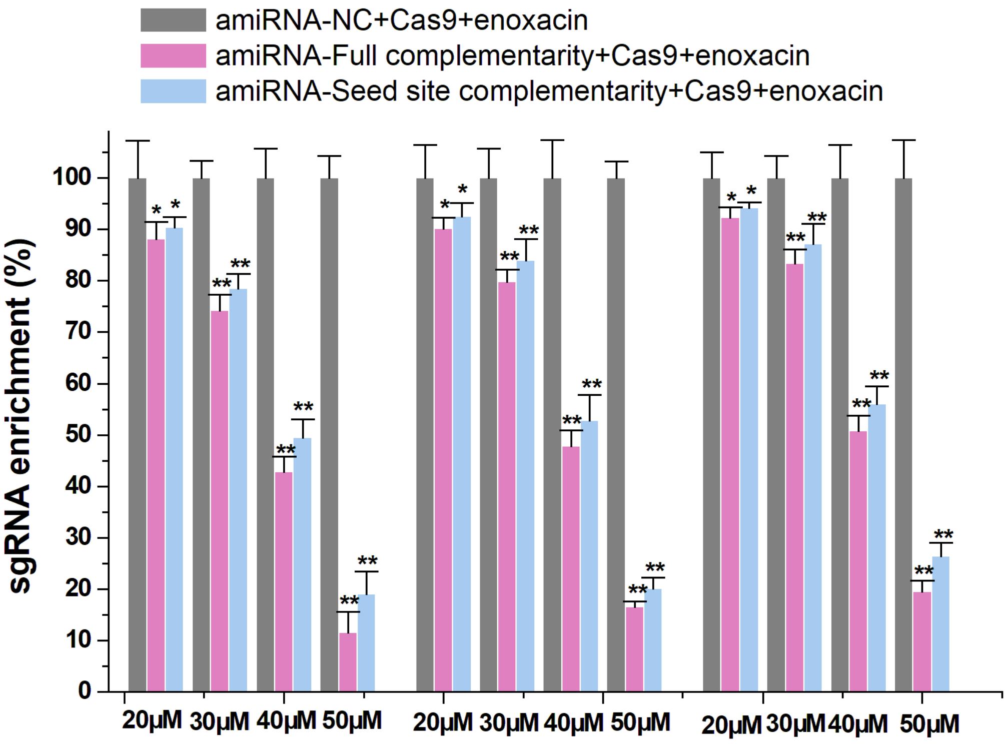

Figure S6. Effects of amiRNAs on the binding of sgRNA to Cas9 in the presence of different concentrations of enoxacin. Cells were co-transfected with sgRNA-Cas9 and amiRNA. After immunoprecipitation, the sgRNA enrichment (%) normalized to the input was measured by qRT-PCR. Reported data are the mean ± SD from five experiments. **P < 0.01, compared with the amiRNA negative control using the paired, one-sided t-test. *P < 0.05, compared with the amiRNA negative control using the paired, one-sided t-test.

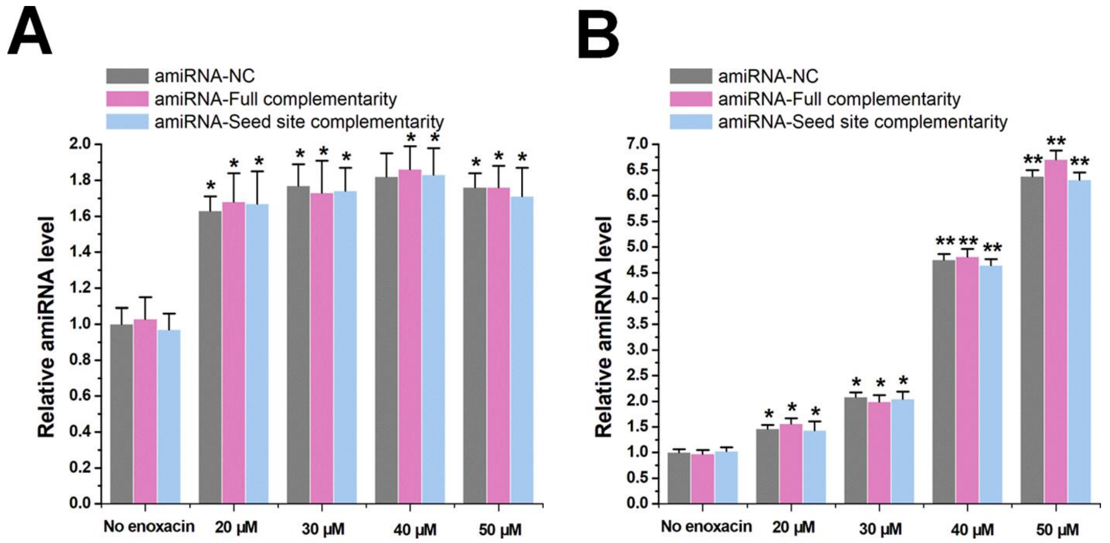

Figure S7. Effect of enoxacin on the processing and loading of amiRNAs onto RISCs. Cells were co-transfected with sgRNA-Cas9 and amiRNA. (**A**) The relative amiRNA level was determined using qRT-PCR after enoxacin treatment. Small nuclear RNA U6 was used as the internal control. *P < 0.05, compared with the amiRNA negative control using the paired, one-sided t-test. (**B**) After immunoprecipitation, the relative amiRNA level, which was normalized to the input, was measured by qRT-PCR. Reported data are the mean ± SD from five experiments. **P < 0.01, compared with the mock control using the paired, one-sided t-test. *P < 0.05, compared with the mock control using the paired, one-sided t-test.

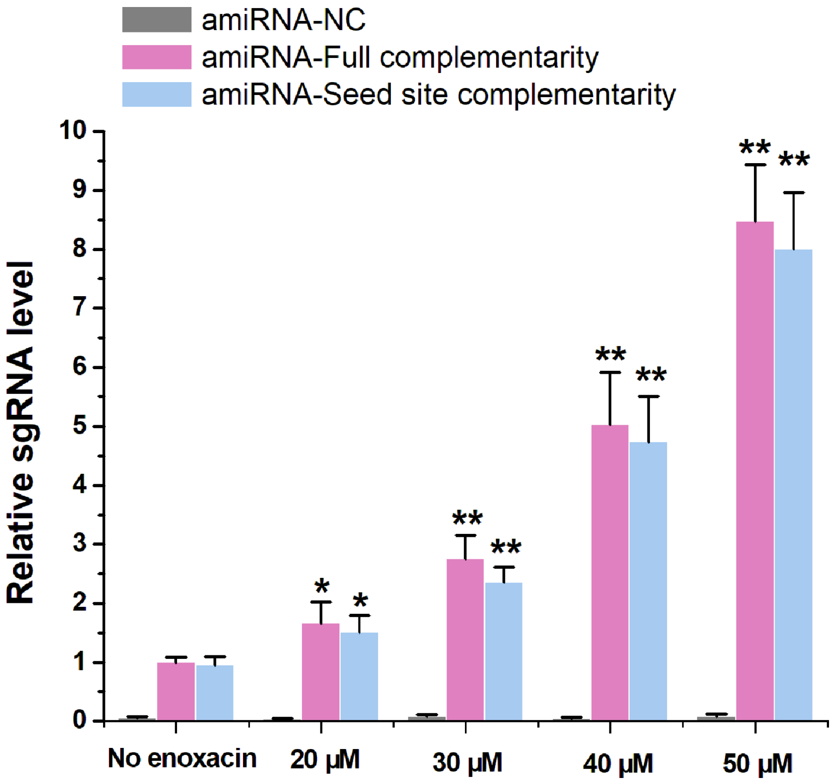

Figure S8. Effect of enoxacin on the relative sgRNA level in miRISCs. Cells were co-transfected with sgRNA-Cas9 and amiRNA. The relative sgRNA level was determined using qRT-PCR after enoxacin treatment. Small nuclear RNA U6 was used as the internal control. Reported data are the mean ± SD from five experiments. *$P < 0.05$, compared with the amiRNA negative control using the paired, one-sided t-test. **$P < 0.01$, compared with the amiRNA negative control using the paired, one-sided t-test.

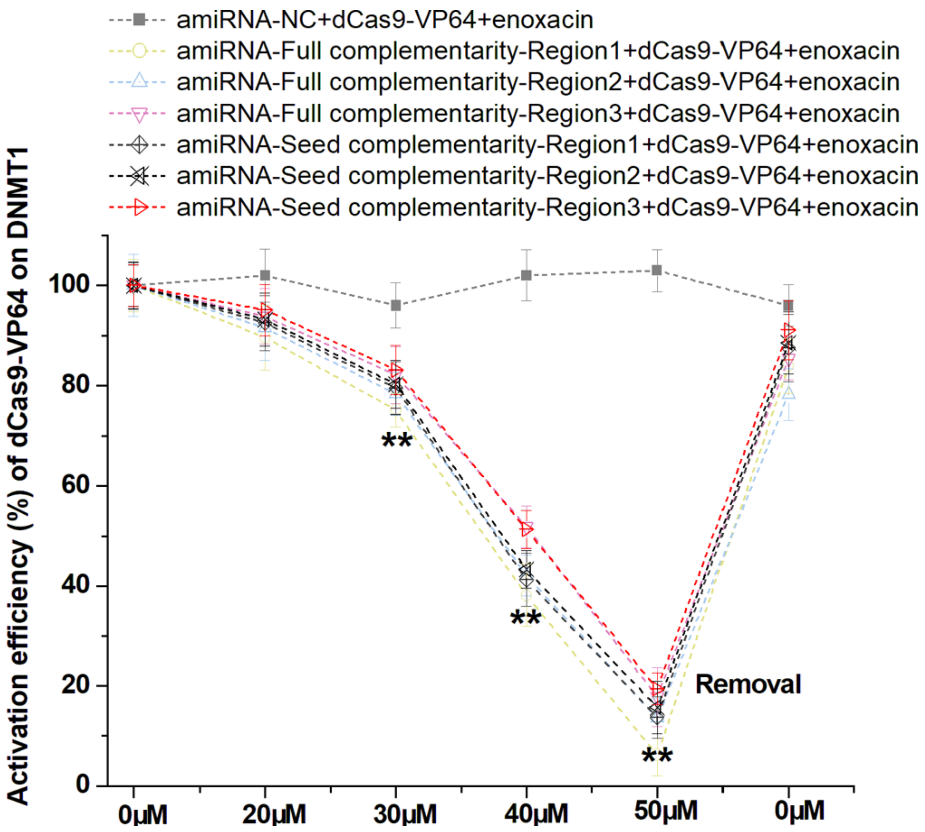

Figure S9. Enoxacin promotes amiRNA-mediated inhibition of CRISPR-dCas9-VP64. Effects of amiRNAs on the expression of *DNMT1* under different concentrations of enoxacin. dCas9-VP64 and *DNMT1* sgRNA were co-transfected into HEK-293T cells, and CRISPRa-mediated regulation of *DNMT1* in response to different doses of enoxacin was measured 48 h after transfection. The dose-effect curve suggested that the inhibition of amiRNA on CRISPRa was enhanced with the increase of enoxacin concentration, and that the effect of CRISPRa was largely restored within 8 h when enoxacin was removed from culture medium. Reported data are the mean ± SD from five experiments. **P < 0.01, compared with the amiRNA negative control using the paired, one-sided t-test.

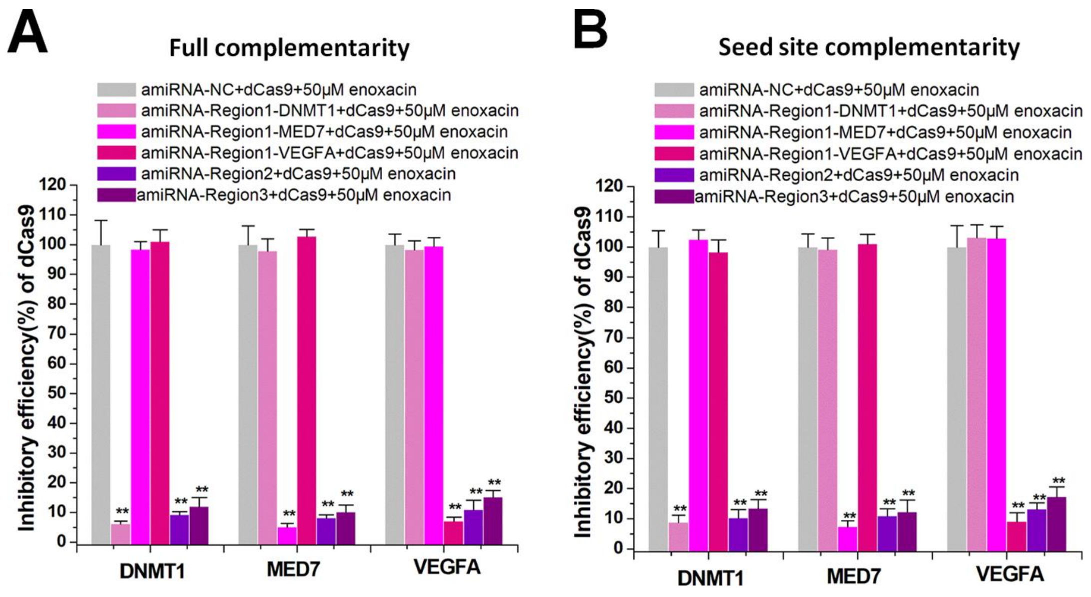

Figure S10. Effects of amiRNAs on the inhibitory efficiency of dCas9 in the presence of 50μM enoxacin. (**A**). Effects of amiRNAs with full sgRNA complementarity on dCas9-mediated transcriptional inhibition. Reported data are the mean ± SD from five experiments. **P < 0.01, compared with the amiRNA negative control using the paired, one-sided t-test. (**B**). Effects of amiRNAs with seed site complementarity on dCas9-mediated transcriptional inhibition. Reported data are the mean ± SD from five experiments. **P < 0.01, compared with the amiRNA negative control using the paired, one-sided t-test.

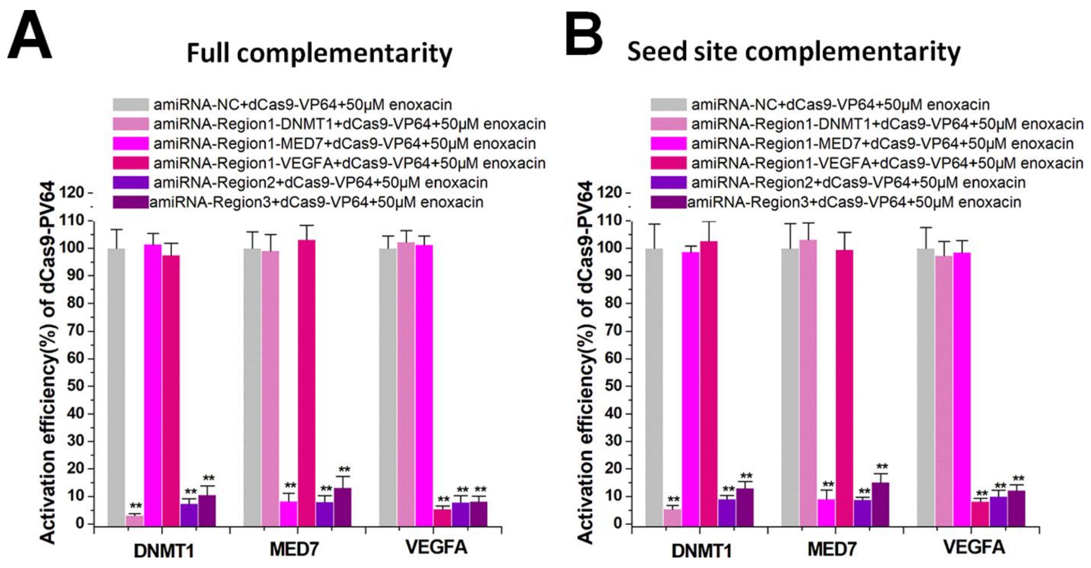

Figure S11. Effects of amiRNAs on the activation efficiency of dCas9-VP64 in the presence of 50μM enoxacin. (**A**). Effects of amiRNAs with full sgRNA complementarity on dCas9-VP64-mediated transcriptional activation. Reported data are the mean ± SD from five experiments. **P < 0.01, compared with the amiRNA negative control using the paired, one-sided t-test. (**B**). Effects of amiRNAs with seed site complementarity on dCas9-VP64-mediated transcriptional activation. Reported data are the mean ± SD from five experiments. **P < 0.01, compared with the amiRNA negative control using the paired, one-sided t-test.

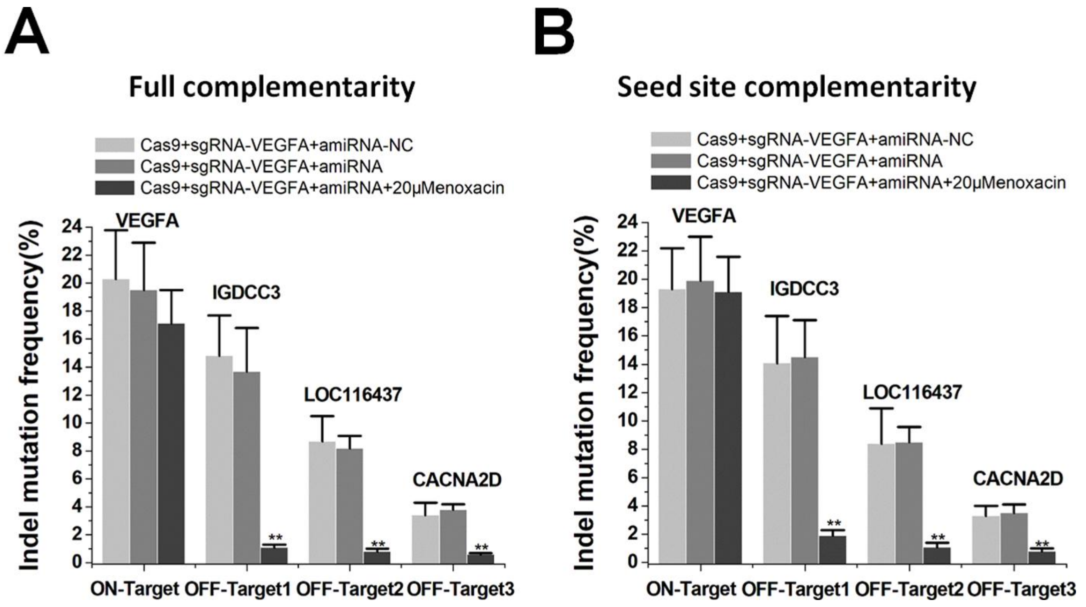

Figure S12. Reducing off-target effects by amiRNAs in the presence of 20μM enoxacin. (**A**) Effects of amiRNAs with full sgRNA complementarity on sgRNA-*VEGFA*. Data are the mean ± SD from five experiments. **P < 0.01, compared with the negative control, determined with a paired, one-sided *t*-test. (**B**) Effects of amiRNAs with seed site complementarity on sgRNA-*VEGFA*. Data are the mean ± SD from five experiments. **P < 0.01, compared with the negative control, determined with a paired, one-sided *t*-test.

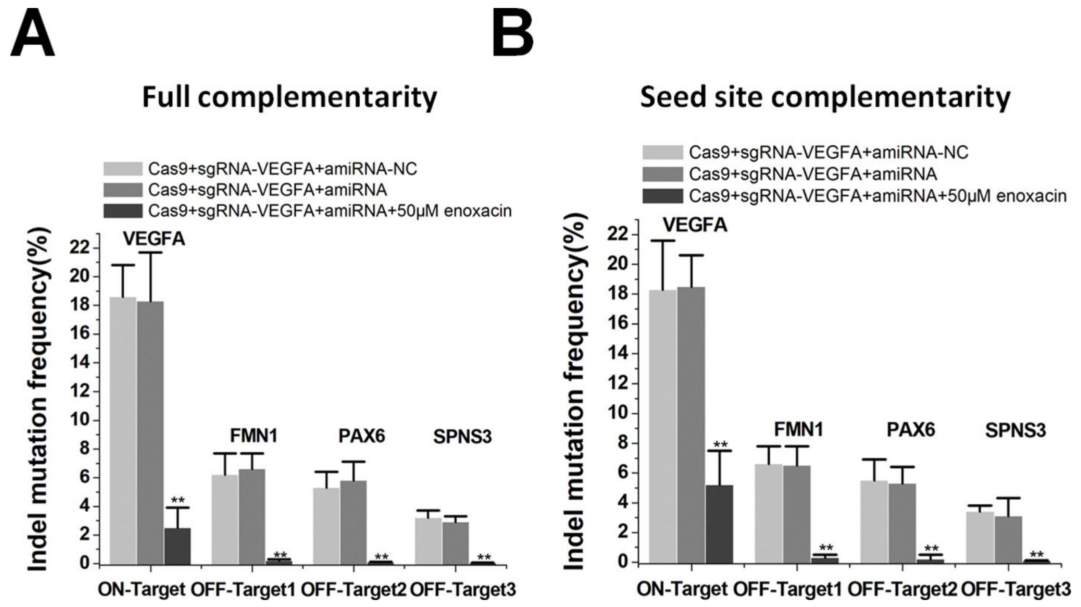

Figure S13. Reducing sgRNA activities at both on-target and off-target sites by amiRNA in the presence of 50μM enoxacin. (**A**) Effects of amiRNAs with full sgRNA complementarity on sgRNAs. Data are the mean ± SD from five experiments. **P < 0.01, compared with the negative control, determined with a paired, one-sided *t*-test. (**B**) Effects of amiRNAs with seed site complementarity on sgRNA. Data are the mean ± SD from five experiments. **P < 0.01, compared with the negative control, determined with a paired, one-sided *t*-test.

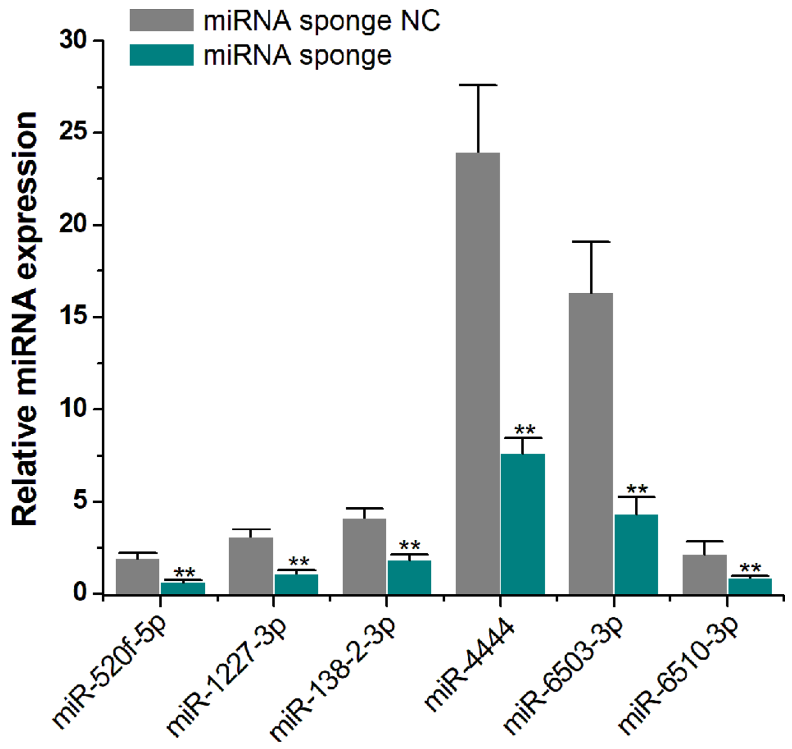

Figure S14. The effect of miRNA sponge on the expression of different miRNAs in HEK-293T cells. The miRNA level was measured by qRT-PCR. U6 was used as an internal control. Data are the mean ± SD from five experiments. **P < 0.01, compared with the negative control, determined with a paired, one-sided *t*-test.

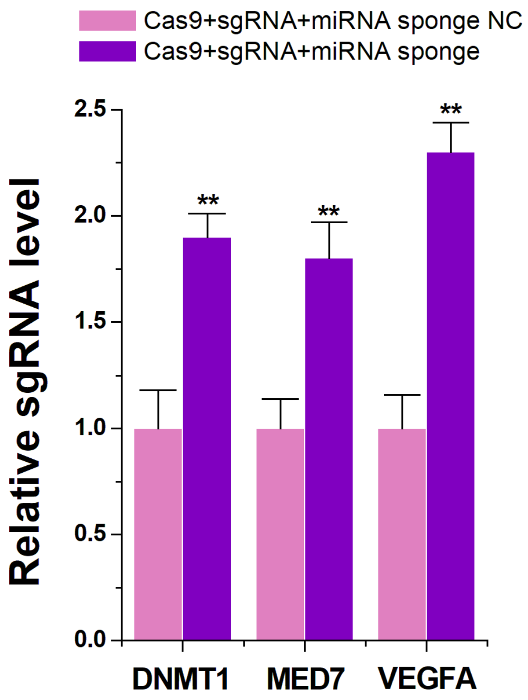

Figure S15. The effect of miRNA sponge on the expression of sgRNAs. Cells were co-transfected with sgRNA-Cas9 and miRNA sponge or negative control sponge. The sgRNA level was measured by qRT-PCR. Small nuclear RNA U6 was used as the internal control. Reported data are the mean ± SD from five experiments. $^{**}P < 0.01$, compared with the sponge negative control using the paired, one-sided t-test.

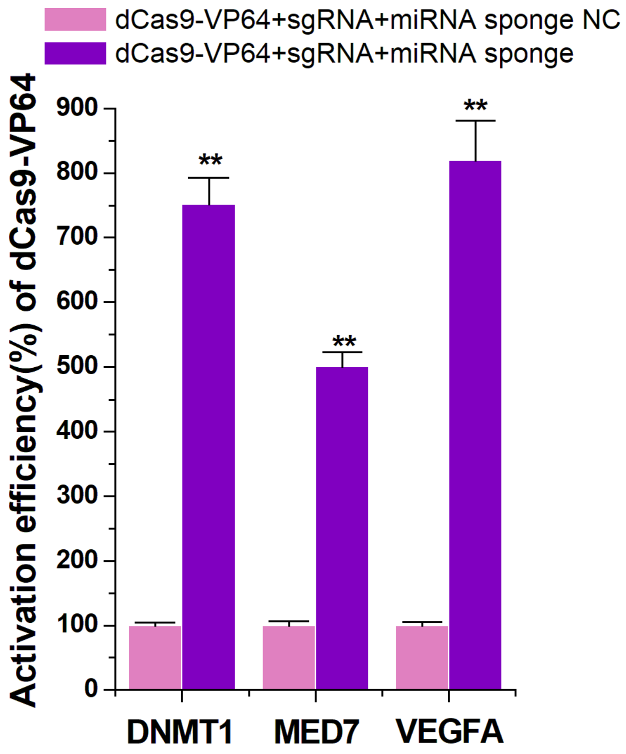

Figure S16. The transcriptional activation efficiency of dCas9-VP64 regulated by the miRNA sponge. HEK-293T cells were transfected with plasmids encoding the dCas9-VP64 and the miRNA sponge. The transcriptional activation efficiency for each gene was determined 48h after transfection. **P < 0.01, compared with the miRNA sponge control, one-sided t-test. Data are the mean ± SD from five experiments.

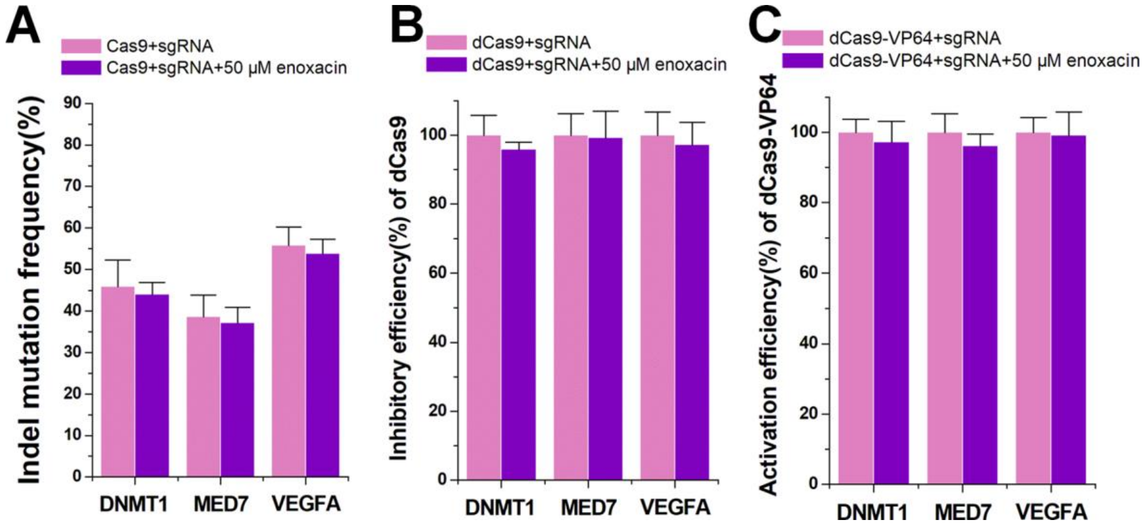

Figure S17. Effects of 50μM enoxacin on the functions of CRISPR systems. (**A**) Effects of 50μM enoxacin on Cas9-mediated gene cleavage efficiency. Data are the mean ± SD from five experiments. (**B**) Effects of 50μM enoxacin on dCas9-mediated transcriptional repression efficiency. Data are the mean ± SD from five experiments. (**C**) Effects of 50μM enoxacin on dCas9-VP64-mediated transcriptional activation efficiency. Data are the mean ± SD from five experiments.

Table S1. The cDNA sequences of the engineered amiRNAs used in this study.

Name	Sequence
pre-amiRNA	AAGGTATATTGCTGTTGACAGTGAGCGCANNNNNNNNNNNNNNNNNNNNNATAGTGAAGCCACAGATGTATNNNNNNNNNNNNNNNNNNNNNTTTGCCTACTGCCTCG
pre-amiRNA-sgRNA-NC (full complementarity)	AAGGTATATTGCTGTTGACAGTGAGCGCAGTACGTTCTCTATCACTGATAATAGTGAAGCCACAGATGTATTATCAGTGATAGAGAACGTACTTTGCCTACTGCCTCG
pre-amiRNA-sgRNA-NC (seed complementarity)	AAGGTATATTGCTGTTGACAGTGAGCGCATGCATCGTCTCGCTACTGATCATAGTGAAGCCACAGATGTATG**ATCAGT**AGCGAGACGATGCATTTGCCTACTGCCTCG
pre-amiRNA-sgRNA-*DNMT1*(Cas9) (full complementarity)	AAGGTATATTGCTGTTGACAGTGAGCGCAGACATCGTCGGGCAGCGAGAATAGTGAAGCCACAGATGTATTCTCGCTGCCCGACGATGTCTTTGCCTACTGCCTCG
pre-amiRNA-sgRNA-*DNMT1*(Cas9) (seed complementarity)	AAGGTATATTGCTGTTGACAGTGAGCGCATCTGCATCAATATAGCGAGCATAGTGAAGCCACAGATGTATG**CTCGCT**ATATTGATGCAGATTTGCCTACTGCCTCG
pre-amiRNA-sgRNA-*DNMT1*(dCas9) (full complementarity)	AAGGTATATTGCTGTTGACAGTGAGCGCAGACATCGTCGGGCAGCGAGAATAGTGAAGCCACAGATGTATTCTCGCTGCCCGACGATGTCTTTGCCTACTGCCTCG
pre-amiRNA-sgRNA-*DNMT1*(dCas9) (seed complementarity)	AAGGTATATTGCTGTTGACAGTGAGCGCATCTGCATGTAAATAGCGAGCATAGTGAAGCCACAGATGTATG**CTCGCT**ATTTACATGCAGATTTGCCTACTGCCTCG
pre-amiRNA-sgRNA-*DNMT1*(dCas9-VP64) (full complementarity)	AAGGTATATTGCTGTTGACAGTGAGCGCAGGGAACACGCATGCGCAAGGATAGTGAAGCCACAGATGTATCCTTGCGCATGCGTGTTCCCTTTGCCTACTGCCTCG

pre-amiRNA-<br>sgRNA-*DNMT1*(dCas9-<br>VP64)<br>(seed complementarity)	AAGGTATATTGCTGTTGACAGTGAGCGCATTT<br>CCTCTATGCTCGCAAGTATAGTGAAGCCACAG<br>ATGTATA**CTTGCG**AGCATAGAGGAAATTTGCC<br>TACTGCCTCG
pre-amiRNA-<br>sgRNA-*MED7*(Cas9)<br>(full complementarity)	AAGGTATATTGCTGTTGACAGTGAGCGCAGAT<br>GATAGCAACAATTGTACATAGTGAAGCCACAG<br>ATGTATGTACAATTGTTGCTATCATCTTTGCCT<br>ACTGCCTCG
pre-amiRNA-<br>sgRNA-*MED7*(Cas9)<br>(seed complementarity)	AAGGTATATTGCTGTTGACAGTGAGCGCATCC<br>TCGCATCCTCATTGTATATAGTGAAGCCACAG<br>ATGTATA**TACAAT**GAGGATGCGAGGATTTGCC<br>TACTGCCTCG
pre-amiRNA-<br>sgRNA-*MED7* (dCas9)<br>(full complementarity)	AAGGTATATTGCTGTTGACAGTGAGCGCAGA<br>AAGACGAAAGACCGCCTTATAGTGAAGCCAC<br>AGATGTATAAGGCGGTCTTTCGTCTTTCTTTG<br>CCTACTGCCTCG
pre-amiRNA-<br>sgRNA-*MED7* (dCas9)<br>(seed complementarity)	AAGGTATATTGCTGTTGACAGTGAGCGCATCC<br>CTCATCCCTCCCGCCTCATAGTGAAGCCACA<br>GATGTATG**AGGCGG**GAGGGATGAGGGATTTG<br>CCTACTGCCTCG
pre-amiRNA-<br>sgRNA-*MED7*<br>(dCas9-VP64)<br>(full complementarity)	AAGGTATATTGCTGTTGACAGTGAGCGCA<br>GAATAGAGGAGTGATAAGACATAGTGAAGCC<br>ACAGATGTATGTCTTATCACTCCTCTATTCTTT<br>GCCTACTGCCTCG
pre-amiRNA-<br>sgRNA-*MED7*<br>(dCas9-VP64)<br>(seed complementarity)	AAGGTATATTGCTGTTGACAGTGAGCGCA<br>TGGCGTCTTGTCTATAAGATATAGTGAAGCCA<br>CAGATGTATA**TCTTAT**AGACAAGACGCCATTT<br>GCCTACTGCCTCG
pre-miRNA-<br>sgRNA-*VEGFA*(Cas9)<br>for Fig.3<br>(full complementarity)	AAGGTATATTGCTGTTGACAGTGAGCGCA<br>GCAGCCCCCGCATCGCATCAATAGTGAAGCC<br>ACAGATGTATTGATGCGATGCGGGGGCTGCT<br>TTGCCTACTGCCTCG
pre-miRNA-<br>sgRNA-*VEGFA*(Cas9)	AAGGTATATTGCTGTTGACAGTGAGCGCA<br>TTCTAATTTATGCCGCATCCATAGTGAAGCCA

for Fig.3<br>(seed complementarity)	CAGATGTATG**GATGCG**GCATAAATTAGAATTTGCCTACTGCCTCG
pre-miRNA-sgRNA-*VEGFA*(Cas9)<br>for Fig.5<br>(full complementarity)	AAGGTATATTGCTGTTGACAGTGAGCGCAGACCCCCTCCACCCCGCCTCATAGTGAAGCCACAGATGTATGAGGCGGGGTGGAGGGGGTCTTTGCCTACTGCCTCG
pre-miRNA-sgRNA-*VEGFA*(Cas9)<br>for Fig.5<br>(seed complementarity)	AAGGTATATTGCTGTTGACAGTGAGCGCATCAATTTCAACTTCCGCCTTATAGTGAAGCCACAGATGTATA**AGGCGG**AAGTTGAAATTGATTTGCCTACTGCCTCG
pre-miRNA-sgRNA-*VEGFA*(Cas9)<br>for Fig.S12<br>(full complementarity)	AAGGTATATTGCTGTTGACAGTGAGCGCAGGGTGGGGGGAGTTTGCTCCATAGTGAAGCCACAGATGTATGGAGCAAACTCCCCCCACCCTTTGCCTACTGCCTCG
pre-miRNA-sgRNA-*VEGFA*(Cas9)<br>for Fig.S12<br>(seed complementarity)	AAGGTATATTGCTGTTGACAGTGAGCGCATTTCTTAATTCTCTTGCTCTATAGTGAAGCCACAGATGTATA**GAGCAA**GAGAATTAAGAAATTTGCCTACTGCCTCG
pre-miRNA-sgRNA-*VEGFA* (dCas9)<br>(full complementarity)	AAGGTATATTGCTGTTGACAGTGAGCGCAGAGCAATCTCCCCAAGCCGTATAGTGAAGCCACAGATGTATACGGCTTGGGGAGATTGCTCTTTGCCTACTGCCTCG
pre-miRNA-sgRNA-*VEGFA* (dCas9)<br>(seed complementarity)	AAGGTATATTGCTGTTGACAGTGAGCGCATCATGGCTGAATTAAGCCGCATAGTGAAGCCACAGATGTATG**CGGCTT**AATTCAGCCATGATTTGCCTACTGCCTCG
pre-miRNA-sgRNA-*VE2GFA*(dCas9-VP64)<br>(full complementarity)	AAGGTATATTGCTGTTGACAGTGAGCGCAGAAAATTACCCATCCGCCCCATAGTGAAGCCACAGATGTATGGGGCGGATGGGTAATTTTCTTTGCCTACTGCCTCG
pre-miRNA-sgRNA-*VEGFA*(dCas9-VP64)<br>(seed complementarity)	AAGGTATATTGCTGTTGACAGTGAGCGCATGGCCGGCTTTGCCCGCCCTATAGTGAAGCCACAGATGTATA**GGGCGG**GCAAAGCCGGCCATTTGCCTACTGCCTCG

pre-miRNA-NC	AAGGTATATTGCTGTTGACAGTGAGCGCATCTCCACGCGCAGTACATTTATAGTGAAGCCACAGATGTATAAATGTACTGCGCGTGGAGATTTGCCTACTGCCTCG

Note: The shadowed part is the target sequence of miRNA. The underlined part is the miRNA mature sequence, and the bold part is the seed sequence of miRNA. The miRNA negative control (NC) sequence is not complementary to any known targets in mammalian cells. The seed sequence between the amiRNA with full sgRNA complementarity targeting a sgRNA region and the amiRNA with seed site complementarity targeting the same region is identical.

Table S2. The cDNA sequences of the engineered miRNA sponges used in this study.

miRNA Sponge	miRNA Sponge Sequence
miR-520f-5p	AGAAAGCGCTGAAGTTTAGAGGCTTC<br>AGAAAGCGCTGAAGTTTAGAGGCTTC<br>AGAAAGCGCTGAAGTTTAGAGG
miR-1227-3p	CTGGGGAACCTTGTGGCACGCTTC<br>CTGGGGAACCTTGTGGCACGCTTC<br>CTGGGGAACCTTGTGGCACG
miRM-138-2-3p	AACCCTGGTGGAACGAAATAGCCTTC<br>AACCCTGGTGGAACGAAATAGCCTTC<br>AACCCTGGTGGAACGAAATAGC
miR-4444	CGCCTCGGAA AACTCGAGCTTC<br>CGCCTCGGAA AACTCGAGCTTC<br>CGCCTCGGAA AACTCGAGCTTC<br>CGCCTCGGAA AACTCGAGCTTC<br>CGCCTCGGAA AACTCGAGCTTC<br>CGCCTCGGAA AACTCGAG
miR-6503-3p	GGAGGTCTGACGA CGAGTCCCCTTC<br>GGAGGTCTGACGA CGAGTCCCCTTC<br>GGAGGTCTGACGA CGAGTCCCCTTC<br>GGAGGTCTGACGA CGAGTCCCCTTC<br>GGAGGTCTGACGA CGAGTCCCCTTC<br>GGAGGTCTGACGA CGAGTCCC
miR-6510-3p	CTGCAGGAGCACAAGTCGGTGCTTC<br>CTGCAGGAGCACAAGTCGGTGCTTC<br>CTGCAGGAGCACAAGTCGGTG
NC	AAGTTTTCAGAAAGCTAACACTTC<br>AAGTTTTCAGAAAGCTAACACTTC<br>AAGTTTTCAGAAAGCTAACA

Note: Shadowed parts are bulged sites that are mispaired opposite miRNA positions 9–12. CTTC, linker; AAGTTTTCAGAAAGCTAACA: an negative control sequence not complementary to any known miRNAs.

Table S3. The cDNA sequences of the engineered sgRNA spacers used in this study.

Names	Sequences
sgRNA-NC	GTACGTTCTCTATCACTGATA
sgRNA-*DNMT1*(Cas9)	GACATCGTCGGGCAGCGAGA
sgRNA-*DNMT1*(dCas9)	GACATCGTCGGGCAGCGAGA
sgRNA-*DNMT1*(dCas9-VP64)	GGGAACACGCATGCGCAAGG
sgRNA-*MED7*(Cas9)	GATGATAGCAACAATTGTAC
sgRNA-*MED7* (dCas9)	GAAAGACGAAAGACCGCCTT
sgRNA- *MED7* (dCas9-VP64)	GAATAGAGGAGTGATAAGAC
sgRNA-*VEGFA*(Cas9) for Fig.3	GCAGCCCCCGCATCGCATCA
sgRNA-*VEGFA*(Cas9) for Fig.5	GACCCCCTCCACCCCGCCTC
sgRNA-*VEGFA*(Cas9) for Fig.S12	GGGTGGGGGGAGTTTGCTCC
sgRNA-*VEGFA* (dCas9)	GAGCAATCTCCCCAAGCCGT
sgRNA-*VEGFA*(dCas9-VP64)	GAAAATTACCCATCCGCCCC

Table S4. Primer sequences used in real-time quantitative PCR.

Names	Sequences
Cas9-F	CAGATTCGCCTGGATGACCA
Cas9-R	ATCCGCTCGATGAAGCTCTG
sgRNA-F (Specific)	The same sequence as the spacer region for each sgRNA
sgRNA-R (Universal)	TTGCACCGACTCGGTG
VEGF-F	ACAGACACCGCTCCTAGCCC
VEGF-R	CGAGAACAGCCCAGAAGTTGG
DNMT1-F	GAGGAGGGCTACCTGGCTAA
DNMT1-R	GCTTAGCCTCTCCATCGGAC
MED7-F	ATCCGCCCTTTGGAAAGTCAG
MED7-R	TGGTGGGGTCGGTATTCATTT
Apoa1-F	GACAGCGGCAGAGACTATGTGT
Apoa1-R	AGGAGATTCAGGTTCAGCTGTTG
GAPDH-F	CGCTCTCTGCTCCTCCTGTTC
GAPDH-R	ATCCGTTGACTCCGACCTTCAC
